# Extracellular vesicles from normal tissues orchestrate the homeostasis of macrophages and attenuate inflammatory injury of sepsis

**DOI:** 10.1002/btm2.10609

**Published:** 2023-10-14

**Authors:** Xinyu Ge, Qingshu Meng, Xuan Liu, Shanshan Shi, Xuedi Geng, Enhao Wang, Mimi Li, Xiaoxue Ma, Fang Lin, Qianqian Zhang, Yinzhen Li, Lunxian Tang, Xiaohui Zhou

**Affiliations:** ^1^ Research Center for Translational Medicine, Shanghai East Hospital Tongji University School of Medicine Shanghai China; ^2^ Shanghai Heart Failure Research Center, Shanghai East Hospital Tongji University School of Medicine Shanghai China; ^3^ Department of thoracic Surgery, Shanghai East Hospital Tongji University School of Medicine Shanghai China; ^4^ Department of Internal Emergency Medicine and Critical Care, Shanghai East Hospital Tongji University School of Medicine Shanghai China; ^5^ Department of Respiratory Medicine, Shanghai East Hospital Tongji University, School of Medicine Shanghai China

**Keywords:** extracellular vesicles, immune homeostasis, macrophage, microRNA, sepsis

## Abstract

Extracellular vesicles (EVs) exist throughout our bodies. We recently revealed the important role of intracardiac EVs induced by myocardial ischemia/reperfusion on cardiac injury and dysfunction. However, the role of EVs isolated from normal tissues remains unclear. Here we found that EVs, derived from murine heart, lung, liver and kidney have similar effects on macrophages and regulate the inflammation, chemotaxis, and phagocytosis of macrophages. Interestingly, EV‐treated macrophages showed LPS resistance with reduced expressions of inflammatory cytokines and enhanced phagocytic activity. Furthermore, we demonstrated that the protein content in EVs contributed to the activation of inflammation, while the RNA component mainly limited the excessive inflammatory response of macrophages to LPS. The enrichment of miRNAs, including miR‐148a‐3p, miR‐1a‐3p and miR‐143‐3p was confirmed in tissue EVs. These EV‐enriched miRNAs contributed to the inflammation remission in LPS induced macrophages through multiple pathways, including STAT3, P65 and SAPK/JNK. Moreover, administration of both EVs and EV‐educated macrophages attenuated septic injury and cytokine storm in murine CLP models. Taken together, the present study disclosed that EVs from normal tissues can orchestrate the homeostasis of macrophages and attenuate inflammatory injury of sepsis. Therefore, tissue derived EVs or their derivatives may serve as potential therapeutic strategies in inflammatory diseases.

**Translational Impact Statements**

This study firstly confirmed the role of EVs from normal tissues on the homeostasis of macrophages and their efficacy in alleviating inflammatory injury of sepsis, and provided important insights into potential therapeutic strategy using EVs from allogeneic tissues or even heterogenic organs for treating inflammatory diseases. In addition, normal tissue EVs‐enriched miRNAs and the EV‐educated macrophages may be developed as novel therapeutic approaches to attenuate the inflammatory injuries.

## 1 INTRODUCTION

Extracellular vesicles (EVs) are cell‐derived nanosized vesicles that exist throughout the body.^1^ Intercellular communications mediated by EVs play vital roles during pathophysiological processes. Recently, several studies^2^, ^3^, ^4^, ^5^ highlighted the role of tissue EVs in the pathogenesis of diseases. However, little is known about whether EVs from vital tissues/organs under healthy conditions influence disease development.

As vital organs supporting life activities, the heart, lung, liver and kidney contain complex resident cell types such as fibroblasts and endothelial cells. Communications between these cells and immune systems are essential for the dynamic equilibrium of the internal environment in tissues, and EVs in tissues are supposed to be the indispensable mediators for the maintenance of immune homeostasis.^6^ As the frontline surveyors of the immune responses, macrophages exist in almost every tissue in the body, differentiate into a variety of subsets in a microenvironment‐dependent manner,^7^ and orchestrate the homeostasis of tissues. Under healthy conditions, macrophages protect the tissues by clearing invading foreign bodies and/or mounting immune responses. During diseases, macrophages are modified to promote the disease progression involving the regulation of inflammation and the repair of injured tissues. Classically, soluble factors, such as cytokines and chemokines, have been recognized as the main mediators to regulate the function of immune cells.^8^ We recently confirmed that EVs from ischemia/reperfusion (IR) injured heart enhanced proinflammatory polarization of macrophages and aggravated the cardiac dysfunction in murine IR injury models.^2^ However, whether EVs from the unperturbed tissues influence the function of macrophages and shape the fate of infectious diseases remain unclear. EVs can transfer specific molecules such as miRNAs^9^, ^10^ into target cells to regulate their function. Thus, we hypothesized that EVs from healthy tissues may exert unique roles in macrophages and further influence the occurrence and development of diseases.

The present study disclosed the features of EVs derived from mouse heart, lung, liver and kidney. These EVs can regulate the function of macrophages in basic and inflammatory conditions. Moreover, EV‐educated macrophages showed LPS resistance with reduced cytokine storm and enhanced phagocytic activity. Consistently, EV‐treatment attenuated septic injury and inhibit the cytokine storm in cecal ligation and puncture (CLP) induced murine septic models. This study for the first time disclosed that EVs from normal tissues can modulation the homeostasis of macrophages and attenuate inflammatory injury of sepsis, indicating that these EVs can serve as “inflammation rheostat” in inflammatory diseases.

## 2 METHODS

### 2.1 Animals

Animals used in the current study were 8–10 weeks old, male, specific pathogen‐free C57BL/6 mice from SLAC Laboratory Animal Co., Ltd. (Shanghai, China). The mice were bred in the Animal Center of Tongji University with constant temperature (23–24°C), humidity (55 ± 5%), and light (12–12 h light—dark schedule). All the animal experimental procedures were reviewed and approved by the Institutional Animal Care and Use Committee of Tongji University (Number: TJLAC‐019‐149).

### 2.2 Establishment of CLP models and in vivo treatments

Mice were randomly (using computer‐generated random numbers) divided into sham or CLP groups. The CLP model was conducted according to previous study.^11^ Briefly, after anesthesia with pentobarbital (50 mg/kg), the abdominal cavity of the mice was exposed. The cecum was ligated tightly with silk sutures (1/3 of cecum), and then punctured twice with an 18‐gauge needle. After returning the cecum into the abdominal cavity and closing the incision, mice were subcutaneously administrated with 1 ml sterile PBS and warmed on a heating pad until recovery from anesthesia. In the sham group, a similar procedure was conducted without ligation and puncture.

For in vivo EVs treatment experiments, mice were intraperitoneally injected with PBS or EVs (2 × 10^9^/g mouse) 1 day before CLP. For EV‐educated macrophages transfer experiments, 2 × 10^6^ RAW264.7 macrophages receiving different treatments were intraperitoneally injected into mice immediately after the CLP surgery.

### 2.3 Tissue‐EV isolation

The isolation method of EVs from different tissues was referred to our previous study.^2^, ^12^ Briefly, the mice were perfused with 10 ml phosphate‐buffered saline (PBS) and then 10 ml 0.1% type II collagenase (Sigma‐Aldrich, USA) from the left ventricle. The tissues including heart, lung, liver and kidney (around 0.2 g) were then cut into small pieces on the ice and digested in PBS containing 0.1% type II collagenase. After a 30 min‐incubation on a shaker at 37°C, the dissociated tissue was first centrifuged at 300×*g* for 5 min at 4°C and successively centrifuged at 2000×*g* and 10,000×*g* for 10 min at 4°C. After filtration with a 1 μm filter, the supernatant was ultracentrifuged at 120,000×*g* at 4°C for 2 h (Optima L‐100XP Ultracentrifuge, Beckman Coulter). The EVs were obtained after washing with PBS.

### 2.4 Transmission electron microscope (TEM) detection

The fresh‐isolated EVs were fixed with 2.5% glutaraldehyde and loaded onto 200 Mesh carbon‐coated formvar grids for 5 min. EVs were stained with 2% phosphotungstic acid for 5 min at room temperature and then detected with a transmission electron microscope (TEM; Hitachi, HT7700).

### 2.5 Nanoparticle tracking analysis (NTA)

Size distribution and the concentration of EVs were detected using the ZetaView NTA technique by Particle Metrix. The protein quantification of EVs was performed using the BCA assay (Promega, USA).

### 2.6 Cell isolation and LPS treatment

Peritoneal macrophages (PMφs) were isolated by peritoneal lavage after injecting 2 ml 3% thioglycollate medium (Merck KGaA, Darmstadt, Germany) for 3 days. The lavage fluid was centrifuged at 1000 rpm for 5 min, and the pelleted cells were resuspended and cultured in 1640 medium with 10% heat‐inactivated EV‐depleted (ultracentrifuged at 120,000×*g* at 4°C for 4 h) fetal bovine serum (FBS, Gibco, Eggenstein, Germany) and 1% penicillin/streptomycin at 37°C in a humidified atmosphere (5% CO_2_, 95% air). After 2‐h incubation, the non‐adherent cells were washed off. The PMφs (1 × 10^6^/well) were pretreated with EVs for 24 h, LPS (100 ng/ml, Sigma‐Aldrich, USA) or PBS was then added for the indicated time.

### 2.7 Phagocytosis assay

The phagocytosis ability of macrophages was detected using the phagocytosis Assay Kit (Cayman 600540, USA) according to the manufacturer's instructions.

### 2.8 Cell‐migration scratch assay

The PMφ cells were grown in 12‐well plates to ~100% confluence in RPMI‐1640 medium with 10% FBS. Cells were then rinsed with PBS and the monolayer was artificially wounded by scratching across each well using a 200 μl pipette tip. The wells were washed with PBS to remove debris followed by replacement of fresh medium or EV‐containing (10^9^/ml) medium. Images were obtained at time 0, and after 24, 48, 72 h, and the “pseudo” wound area was measured by Image‐Lab software (Bio‐Rad Laboratories). The migration ability of macrophages at the indicated time points was expressed as a percentage of “pseudo” wound area with respect to that at time 0.

### 2.9 Bacteria count determination

The quantification of the bacterial load in the peritoneal lavage fluid (PLF) was performed 1 day after CLP. The peritoneal lavage was performed after injecting sterile PBS into the peritoneal cavity. A total of 10 ml of PLF was collected. After the sample collection, 20 μl PLF with 1:100 and 1:1000 dilutions were plated on LB agar and incubated at 37°C under aerobic conditions for 18 h. Colony‐forming units (CFUs) were counted, and the colony counts of peritoneal wash were calculated. All procedures were performed under sterile conditions.

### 2.10 RNA isolation and quantitative real‐time PCR

Total RNAs from cells, tissues or EVs were extracted using TRIzol reagent (Invitrogen, USA). RNA was converted to cDNA with PrimeScript RT Master Mix (Takara RR036A) and the interested mRNA expression was detected using TB Green Fast qPCR Mix (Takara RR430A). For miRNA reverse transcription, miRcute enhanced miRNA cDNA first‐strand synthesis Kit (Tiangen KR211) was used. The transcript levels of β‐actin and U6 were tested as the internal control for mRNA and miRNA, respectively. All the primers used in the study were listed in Table S1.

### 2.11 Western blot

After indicated treatment, the samples were lysed with RIPA Buffer. Proteins were extracted and loaded on 10% SDS‐PAGE gels, then transferred to PVDF membranes. After blocking for 1 h at room temperature, the membranes were incubated overnight at 4°C with primary antibodies directed to: CD63 (Santa Cruz, sc‐5275, 1:400 dilution), CD9 (Abcam ab92726, 1:1000 dilution), Alix (Abcam ab117600, 1:1000 dilution), TSG101 (Abcam ab125011, 1:000 dilution), STAT3 (CST 4904, 1:000 dilution), p‐STAT3 (CST 9145, 1:000 dilution), P65 (CST 8242, 1:000 dilution), p‐P65 (CST 3033, 1:000 dilution), SAPK/JNK (CST 9252, 1:000 dilution), p‐SAPK/JNK (CST 4668, 1:000 dilution), and β‐actin (CST 3700, 1:000 dilution). After TBST washing, the membranes were incubated with corresponding secondary antibodies (CST 7074 or 7076, 1:1000 dilution) for 1 h, the bands were detected by chemiluminescence (Pierce, USA).

### 2.12 Transfection of miRNA mimics and inhibitors

The RAW264.7 cells and PMφs as indicated in the study were transfected with negative control miRNA or miR‐1a‐3p, miR‐143‐3p, miR148a‐3p mimic or miR‐1a‐3p, miR‐143‐3p, miR148a‐3p inhibitor (synthesized commercially by Sangon Biotech, China) using ribo*FECT* CP Transfection Regent (RiboBio R10035.3, China). For EVs transfection, Lipofectamine RNAiMAX (Invitrogen, USA) was used according to the manufacturer's instructions.

### 2.13 Flow cytometry analysis

After indicated treatments, the PMφs were suspended in PBS and incubated with fluorescence conjugated CD80 and MRC1 antibody (BioLegend, USA) at 4°C for 30 min. For intracellular staining of NOS2, cells were fixed and permeabilized in accordance with manufacturers' instructions (BD Biosciences, USA) before antibody incubation. Macrophages in lung tissues were detected and the detailed methodology can be found in supplemental methods. Data were acquired using a BD FACSCanto II flow cytometer (BD Bioscience, USA) and analyzed with FlowJo‐V10 software (Tree Star, Ashland, OR).

### 2.14 Confocal imaging

EVs were stained with the lipophilic dye 1,1′‐dioctadecyl‐3,3,3′,3′‐tetramethylindocarbocyanine perchlorate (DiI; 10 μM, Beyotime Biotechnology, China) according to the manufacturer's protocol and cultured with PMφs for 24 h. Cells were washed two times with PBS and then fixed with 4% (w/v) paraformaldehyde for 15 min. DAPI (Invitrogen, USA) was used for nuclei staining. Confocal images were obtained using a Leica SP8 System (Leica, Wetzlar, Germany).

### 2.15 H&E staining and immunofluorescence imaging

Mice were sacrificed and then perfused with PBS through the left ventricle for 2 min followed by 4% paraformaldehyde for 5 min. The lungs were excised, and immersion fixed overnight. After paraffin embedding and tissue sections preparation, samples were stained with hematoxylin and eosin (H&E) and observed by light microscopy. For immunofluorescence imaging, tissue sections were rehydrated in PBS for 15 min, permeabilized in 0.2% Triton X‐100 for 45 min and blocked in 1% BSA for 1 h. Antibodies against CD45 and F4/80 (Abcam, USA) were incubated overnight. Next, secondary antibodies with Alexa Fluor 488 and Alexa Fluor 568 (CST, USA) were used. The tissues were imaged using a Leica DM2500 microscope (Leica, Wetzlar, Germany).

### 2.16 Statistical analysis

The data were presented as the mean ± SD and analyzed using Prism 8.0 (GraphPad Software Inc.). The Student's *t*‐test was used for comparison between two groups. Multiple groups comparison was performed with one‐way analysis of variance (ANOVA), followed by the Bonferroni test. Correlation analysis was performed using spearman's rho test. A *p* value <0.05 was considered statistically significant.

## 3 RESULTS

### 3.1 Characterization of the tissue derived EVs

**FIGURE 1:**
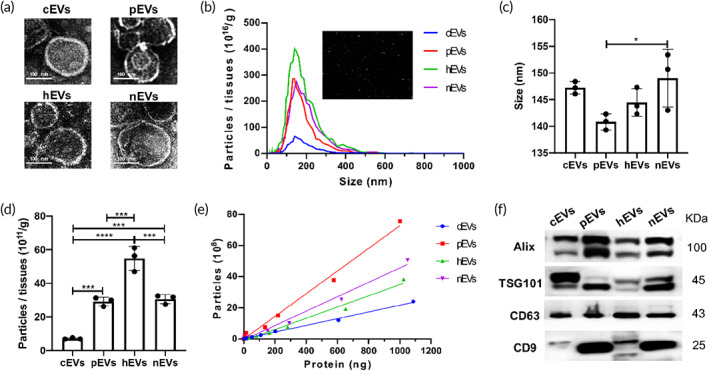
Characterization of the tissue derived EVs. (a) A representative TEM image of EVs derived from the heart (cardiac EVs, cEVs), lung (pulmonary EVs, pEVs), liver (hepatic EVs, hEVs) and kidney (nephritic EVs, nEVs), (bar = 100 nm). (b) The particle size distribution of the EVs was measured using nanoparticle tracking analysis (NTA). (c) The particle size of different tissue derived EVs. (d) Quantification of EVs isolated from different tissues. (e) The linear relationships between particle number and total protein content of EVs. (f) Protein immunoblots of EVs, including four typical exosomal markers (Alix, Tsg101, CD63, and CD9). *p < 0.05; ***p < 0.001; ****p < 0.0001.

The typical morphology of EVs from the heart (cardiac EVs, cEVs), lung (pulmonary EVs, pEVs), liver (hepatic EVs, hEVs) and kidney (nephritic EVs, nEVs) were captured under TEM (Figure 1a). The particle size measured by NTA showed the average diameter of cEVs (147.2 nm), pEVs (140.8 nm), hEVs (143.1 nm), and nEVs (153.3 nm) in Figure 1b,c. The liver contained the largest number of vesicles per gram. Comparatively, the concentration of EVs was less in the heart (Figure 1d). As shown in Figure 1e, linear associations were found in EVs between particle numbers and total protein contents. cEVs contained rich protein components while pEVs possessed the least protein (Figure 1e). Moreover, typical exosome markers including Alix, TSG101 CD63 and CD9 were expressed in this tissue derived EVs (Figure 1f).

### 3.2 EVs from different tissues reshape the functions of macrophages

**FIGURE 2:**
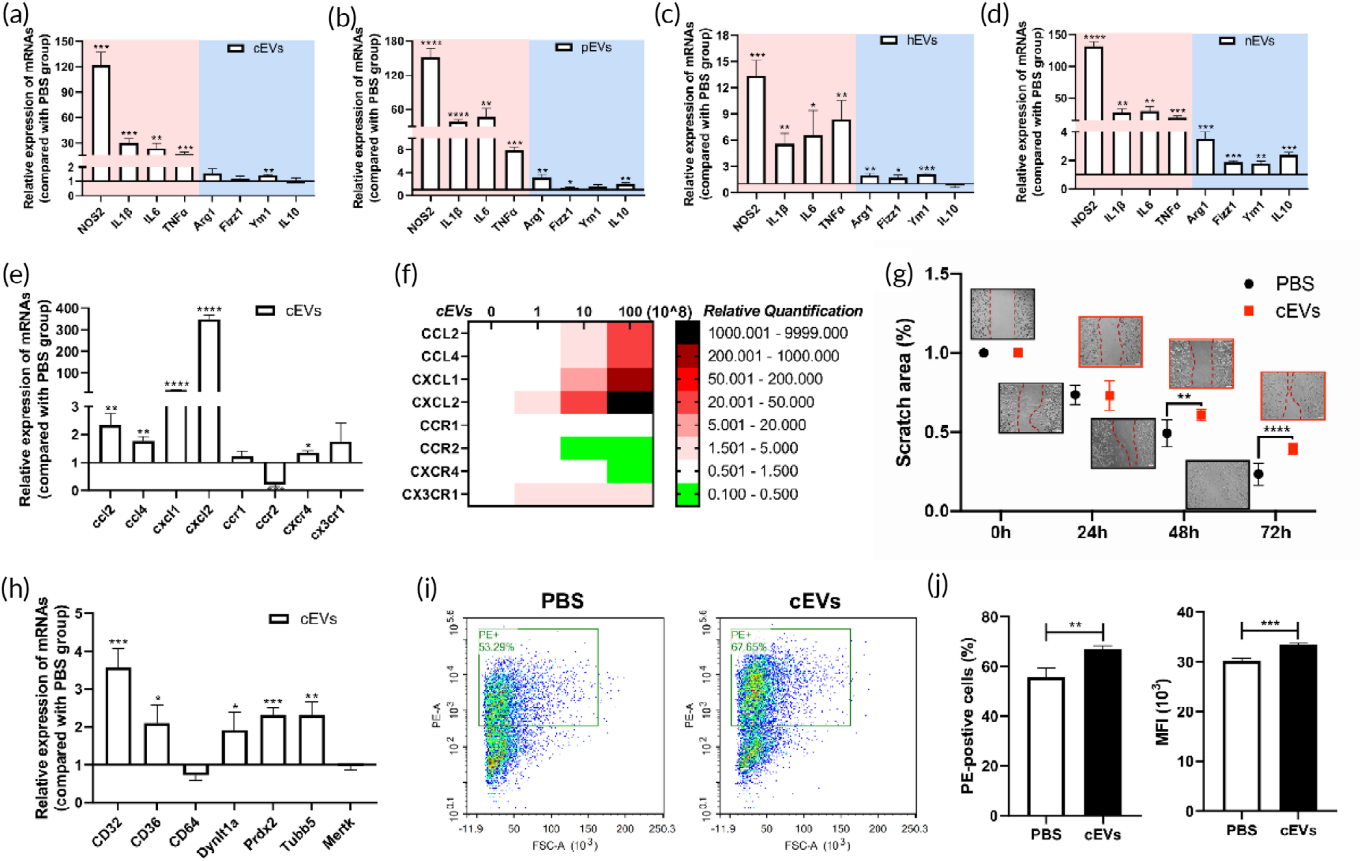
Tissue‐derived EVs reprogramme the function of macrophages. The expressions of M1 and M2 polarization related genes in primary peritoneal macrophages treated with (a) cEVs, (b) pEVs, (c) hEVs and (d) nEVs overnight. (e) The expressions of chemokines (ccl2, ccl4, cxcl1, and cxcl2) and chemokine receptors (ccr1, ccr2, cxcr4, and cx3xr1) in macrophages treated with cEVs. (f) The expressions of chemokines (ccl2, ccl4, cxcl1, and cxcl2) and chemokine receptors (ccr1, ccr2, cxcr4, and cx3xr1) in macrophages treated with gradient concentrations of IR‐EVs overnight. (g) Wound healing test of macrophages treated with PBS or cEVs. (h) The expressions of phagocytosis‐related genes in macrophages treated with cEVs. (i) Phagocytic function of peritoneal macrophages was detected by phagocytosis assay. (j) Statistical results. These data were representative results (*n* = 3) of three repetitions. *p < 0.05; **p < 0.01; ***p < 0.001; ****p < 0.0001.

Macrophages can be regulated by local environmental EVs upon entry into tissues. Our previous study demonstrated that murine cardiac EVs post IR triggered the M1‐like polarization of macrophages and contributed to the pathological process of myocardial injury.^2^ Here, we sought to determine whether tissue EVs affect macrophage performance in basic conditions. Unexpectedly, we found that cEVs significantly enhanced the activation of macrophages with increased expressions of NOS2, IL1β, IL6, and TNFα (Figure 2a **)**. Similar responses also existed in macrophages treated with pEVs, hEVs, and nEVs (Figure 2b—d). Interestingly, unlike typical M1‐polarization inducers (such as LPS), these EVs did not significantly reduce the expressions of anti‐inflammatory factors but even increased their expressions in macrophages (Figure 2a—d). Further, treatment with the tissue EVs (cEVs, for example) increased the expressions of inflammatory pathway related genes including STAT1 and STAT3 (Figure S1A,B), and enhanced their suppressor genes (SOCS1 and SOCS3) as well in macrophages (Figure S1C, D). Chemokines including Ccl2, Ccl4, Cxcl1, and Cxcl2 were all significantly increased at the mRNA level, while the expression of CCR2 was significantly reduced in macrophages treated with cEVs (Figure 2e, f) and other tissue‐derived EVs (Figure S2A–C). Addition of cardiac EVs also inhibited the migration ability of macrophages (Figure 2g). In addition, the expressions of phagocytic‐related genes were elevated in macrophages treated with cEVs (Figures 2h and S3A–D) and other tissue‐derived EVs (Figure S3E–G). Consistently, cEV treatment promoted the phagocytosis activity of macrophages compared to the control (Figure 2i, j).

To further exclude the effect of possible contaminations of the tissue EVs on macrophages, we purified the cEVs using size exclusion chromatography (SEC). Consistently, purified‐EVs showed the similar effects on macrophages with cEVs (Figure S4A–C). Purified‐EV‐treated macrophages showed much higher mRNA expressions of M1 polarized related genes (NOS2, IL1β, IL6, and TNFα), chemokines and chemokines receptors such as CCL4, CXCL2, and CCR1 and phagocytosis related genes (CD32, CD36, and Prdx2) than that in the filtrate group (Figure S4A–C). While the filtrate that may contain non‐EV contaminations only showed little effect on the modulation of macrophages. Therefore, EVs from healthy tissues can modulate the functions of macrophages including the upregulation of cytokine/chemokine expressions and promotion of phagocytic function.

### 3.3 Tissue‐derived EVs limit LPS induced cytokine storm in macrophages

**FIGURE 3:**
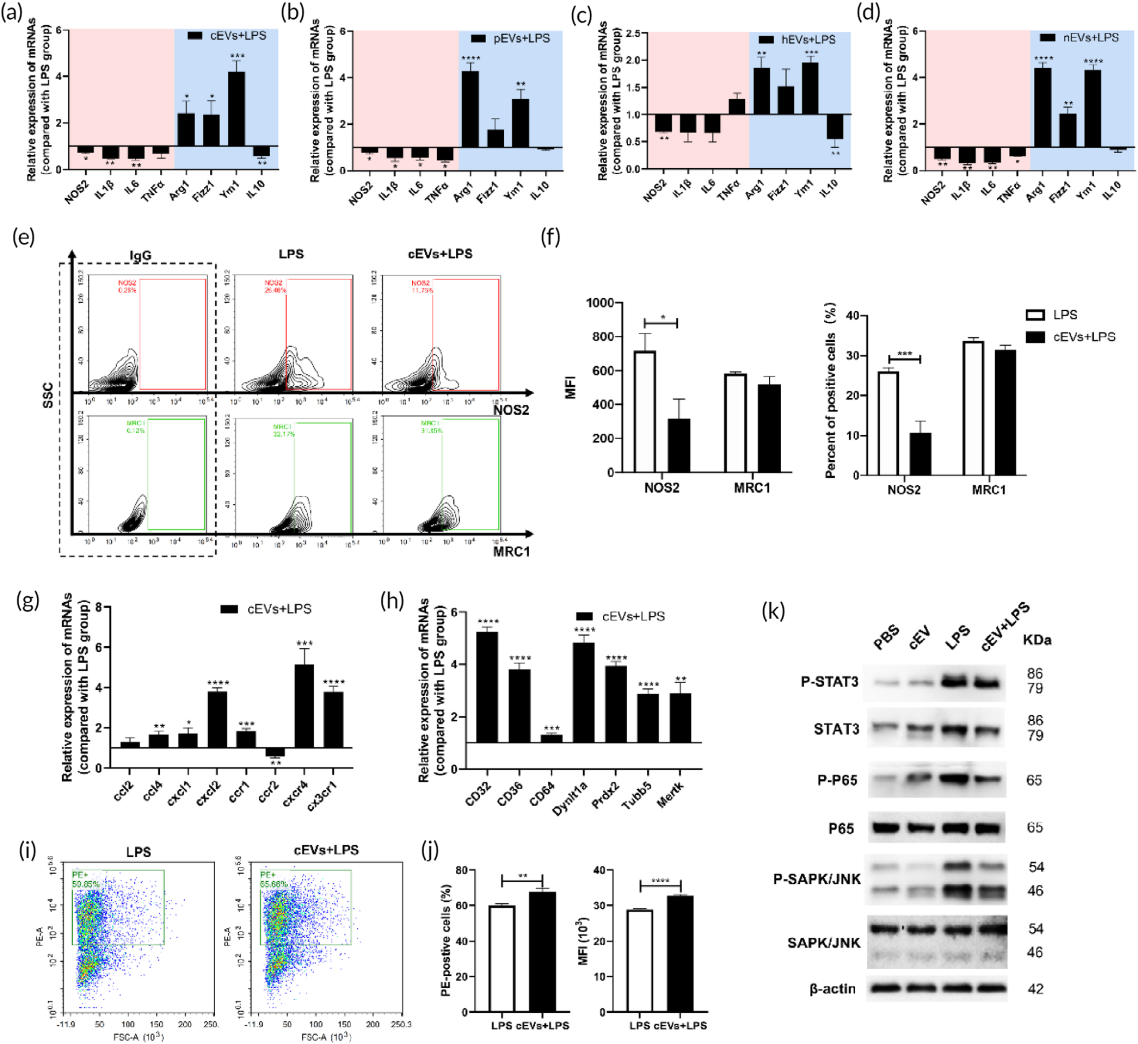
Tissue‐derived EVs modulate the performance of macrophages receiving LPS treatment. Primary peritoneal macrophages were pretreated with (a) cEVs, (b) pEVs, (c) hEVs, and (d) nEVs overnight and then stimulated with LPS for 3 h, the expressions of M1 and M2 polarization related genes were detected. (e) Polarization of macrophages was determined by flow cytometry. (f) Statistical results in (e). (g) The expressions of chemokines (ccl2, ccl4, cxcl1, and cxcl2) and chemokine receptors (ccr1, ccr2, cxcr4, and cx3xr1) in macrophages in (a). (h) The expression of phagocytosis‐related genes in macrophages in (a). (i) The phagocytic function of macrophages in (a) was detected by phagocytosis assay. (j) Statistical results in (i). (k) Western blot of pathway proteins in macrophages in (a). These data were representative results (*n* = 3) of three repetitions. *p < 0.05; **P < 0.01; ***p < 0.001; ****p < 0.0001.

Next, we explored the functional status of the EV‐educated macrophages through LPS stimulation. When stimulated by LPS, EVs pretreated macrophages showed decreased expressions of M1 polarization related genes, including NOS2, IL1β, IL6 and TNFα, and increased expression of M2 polarization related genes, like Arg1 and Ym1 (Figure 3a–d). Flow cytometry analysis further disclosed that EVs pretreatment decreased the proportion of NOS2^+^ (M1 marker) macrophages but did not influence the frequency of MRC1^+^ (M2 marker) cells (Figure 3e,f) in LPS stimulation culture system. Moreover, EVs pretreatment increased the expressions of chemokines, chemokine receptors (Figures 3g and S5) and phagocytic‐related genes (Figures 3h and S6), and enhanced phagocytosis of the macrophage receiving LPS stimulation (Figure 3i, j). Furthermore, the purified‐EVs rather than the filtrate significantly decreased the expression of inflammatory cytokines and increased the expression of chemokines and phagocytic‐related genes (Figure S7A–C) in LPS stimulated macrophages. Then, we checked the key proteins of inflammation‐related pathways. Compared with the control LPS group, EV pretreatment reduced the expression of p‐STAT3/STAT3, p‐P65, and p‐SAPK/JNK (Figure 3k). These results indicated that EVs education restricted the inflammatory cytokine storm and promoted the chemotactic and phagocytic functions of macrophages when stimulated with LPS.

### 3.4 RNAs and proteins in tissue derived EVs differentially regulate the function of macrophages

**FIGURE 4:**
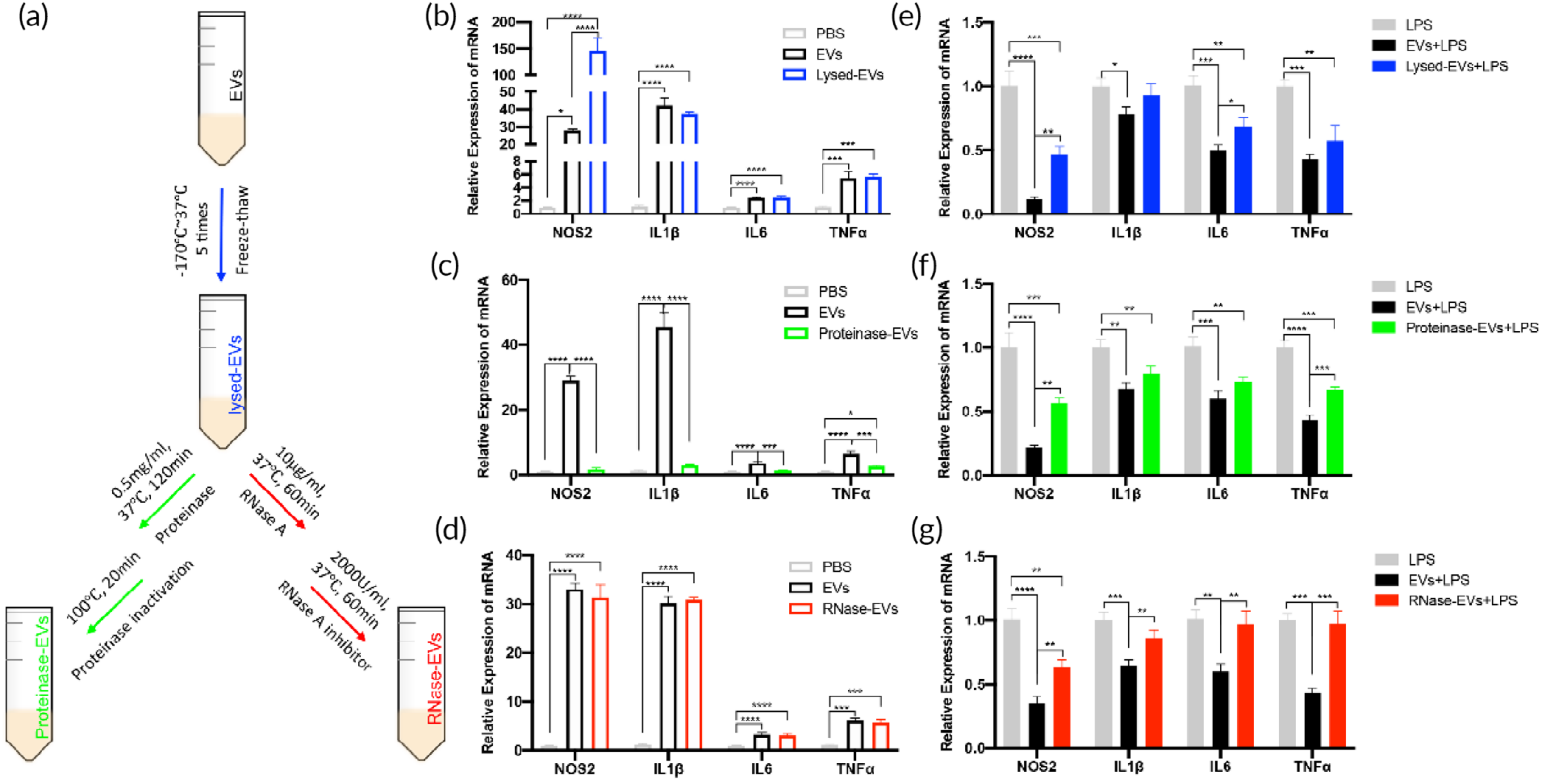
RNAs and proteins in EVs differentially modulate the function of macrophages. (a) Flowchart illustrated the experimental procedures for the removal of proteins or RNAs in cardiac EVs. Primary peritoneal macrophages were used for further experiment. (b) qPCR analysis showed the proinflammatory effect of EVs and lysed‐EVs. (c) qPCR analysis showed the proinflammatory effect of EVs and proteinase‐EVs. (d) qPCR analysis showed the proinflammatory effect of EVs and RNase‐EVs. (e) qPCR analysis showed the effect of EV and lysed‐EV pretreatment on the LPS responses of macrophages. (f) qPCR analysis showed the effect of EV and proteinase‐EV pretreatment on the LPS responses of macrophages. (g) qPCR analysis showed the effect of EV and RNase‐EV pretreatment on the LPS responses of macrophages. These data were representative results (*n* = 3) of three repetitions. *p < 0.05; **p < 0.01; ***p < 0.001; ****p < 0.0001.

EVs are known to regulate the function of target cells by delivering active molecules such as RNA and proteins. To identify whether proteins or RNAs are responsible for the inhibition of LPS‐induced cytokine storm in macrophages, the experimental procedures for removal of proteins or RNAs in cEVs were designed according to a previous study^13^ (Figure 4a). Firstly, we found that EV destruction had similar effects with the untouched EVs on the cytokine production in primary macrophages without LPS stimulation (Figure 4b). Protein degradation in EVs neutralized the pro‐inflammatory effect of EVs (Figure 4c), while RNA degradation failed to change the pro‐inflammatory effect of EVs on primary macrophages in basic conditions (Figure 4d). Secondly, the lysed‐EVs preserved the inflammatory inhibition effect on LPS‐induced macrophages, although such inhibition effect was not as strong as that of the intact ones (Figure 4e). Protein degradation in EVs still comparably reduced the inflammatory response of macrophages treated with LPS (Figure 4f), however, RNA degradation in EVs diminished the inflammatory suppression in macrophages treated with LPS compared to the intact EVs (Figure 4g). These results suggested that the protein in EVs is crucial to the inflammatory activation of macrophages, while the RNA component can limit the excessive inflammatory response of macrophages to LPS.

### 3.5 MicroRNAs are enriched in tissue‐derived EVs and can be transferred to macrophages

**FIGURE 5:**
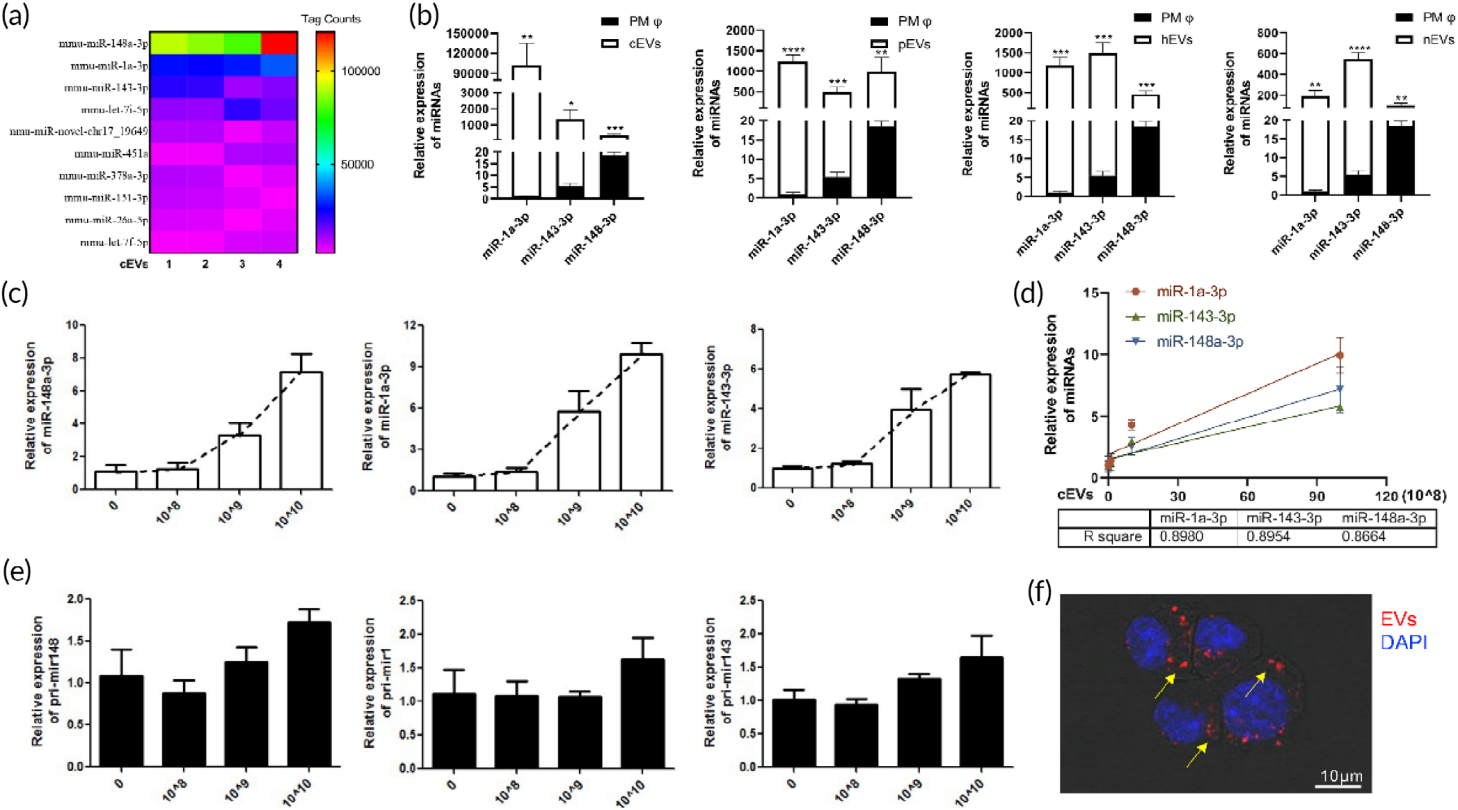
The EV‐enriched miRNAs could be transferred to macrophages. (a) Heat map showed the highly (top 10) expressed miRNAs in cardiac EVs. (b) qPCR analysis showed the expressions of top three miRNAs including miR‐1a‐3p, miR‐143a‐3p and miR‐148‐3p in the macrophage and different tissue derived EVs. (c) A dose‐dependent increase of miR‐148‐3p, miR‐1a‐3p, and miR‐143a‐3p expressions in macrophages receiving cEV treatment. X‐axis showed the numbers of EVs. (d) A positive correlation between EV concentration and the expressions of miR‐1a‐3p, miR‐143a‐3p, and miR‐148‐3p in the treated macrophages. (e) The expressions of pri‐mir148, pri‐mir1, and pri‐mir143 in macrophages treated with gradient concentrations of cEVs for 24 h. X‐axis showed the numbers of EVs. (f) Confocal images showed the uptake of cEVs by macrophages after coculture with DiI‐labeled EVs for 24 h. These data were representative results (*n* = 3) of three repetitions. *p < 0.05; **p < 0.01; ***p < 0.001; ****p < 0.0001.

Our recent report confirmed the abundance of miRNAs in cardiac EVs by RNA sequencing.^2^ The top three miRNAs in cEVs were miR‐148a‐3p, miR‐1a‐3p, and miR‐143‐3p (Figure 5a). Compared with macrophages, these miRNAs were significantly enriched in cEVs (Figure 5b). Interestingly, we also found marked enrichment of these miRNAs in pEVs, hEVs and nEVs (Figure 5b). Moreover, these miRNAs were significantly up‐regulated in macrophages after cocultured with cEVs (Figure 5c), and the concentration of EVs was positively correlated with their miRNA expressions (Figure 5d). While cEV treatment did not increase the expression of the pri‐miRNAs (Figure 5e). Confocal microscopy revealed that the EVs can be uptaken by macrophages, which consistently embodied their carrier property (Figure 5f). Collectively, we identified that miRNAs, including miR‐148a‐3p, miR‐1a‐3p, and miR‐143‐3p, were enriched in different tissue EVs and can be transferred into macrophages.

### 3.6 EV‐enriched miRNAs contribute to the inflammation remission in LPS induced macrophages

**FIGURE 6:**
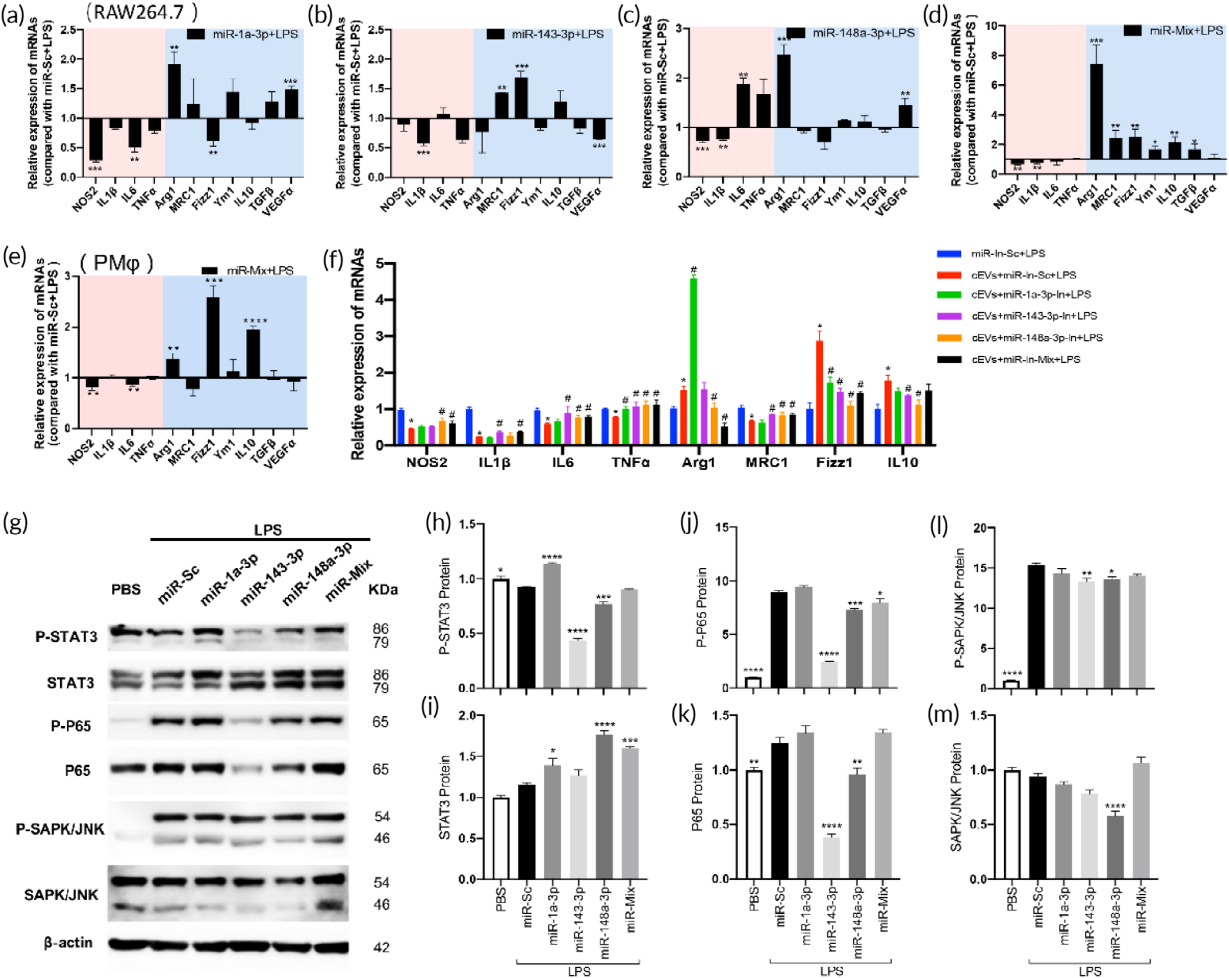
The EV‐enriched miRNAs inhibited the inflammatory response in LPS treated macrophages. The macrophage RAW264.7 were transfected with miRNA mimics of (a) miR‐1a‐3p, (b) miR‐143‐3p, (c) miR‐148a‐3p, and (d) their mix, and then treated with LPS for 3 h. The expression of M1 and M2 related genes was detected by qPCR. (e) Peritoneal macrophages (PMφ) were transfected with the miRNA mix and then treated with LPS for 3 h. The expression of M1 and M2 related genes was detected by qPCR. (f) qPCR analysis showed the effect of cEVs transfected with the miRNA‐inhibitors and their mix on the inflammatory activation of LPS‐treated macrophage RAW264.7. (g) Western blot of pathway proteins in macrophages receiving miRNA transfection and then treated with LPS for 3 h. Statistical results in (g) including the expressions of (h) p‐STAT3, (i) STAT3, (j) p‐P65, (k) P65, (l) p‐SAPK/JNK, (m) SAPK/JNK were calculated. miR‐In‐Sc, miRNA inhibitor scramble; miR‐1a‐3p‐In, miR‐1a‐3p Inhibitor; miR‐143‐3p‐In, miR‐143‐3p Inhibitor; miR‐148a‐3p‐In, miR‐148a‐3p Inhibitor; These data were representative results (*n* = 3) of three repetitions. *p < 0.05 vs. miR‐In‐Sc + LPS; ^#^ *p* < 0.05 vs. cEVs+miR‐In‐Sc + LPS.

Next, miRNA mimics and inhibitors were used to confirm the effect of these highly enriched miRNAs on macrophages. Firstly, we found that transfection of each miRNA and their mix decreased the expressions of multiple inflammatory cytokines (Figure S8A—E) and inhibited the M1 polarization induced by LPS (Figure 6a–e). Then, we performed transfection of miRNA mimics and inhibitors into cEVs as the representative tissue‐EVs. Transfection of each miRNA‐inhibitors or their mix upregulated the EV‐induced proinflammatory effect slightly compared to the EVs with miR‐In‐Sc (Figure S8F). However, addition of miRNA‐inhibitors or their mixtures did partly neutralize the anti‐inflammatory effect of EVs on LPS induced macrophages (Figure 6f). Moreover, addition of these miRNA mimics affected different signaling pathway proteins (Figure 6g–m). We found significant inhibition of p‐STAT3 by miR‐143‐3p and miR‐148a‐3p (Figure 6h), p‐P65 and P65 by miR‐143‐3p and miR‐148a‐3p (Figure 6j, k), p‐SAPK/JNK by miR‐143‐3p and miR‐148a‐3p (Figure 6l), and SAPK/JNK by miR‐148a‐3p (Figure 6m). These results revealed that miR‐1a‐3p, miR‐143‐3p, and miR‐148a‐3p in EVs can regulate the inflammatory responses induced by LPS in macrophages and these effects were mediated by distinct pathways.

### 3.7 Administration of tissue derived EVs attenuate the inflammatory injury in murine sepsis models

**FIGURE 7:**
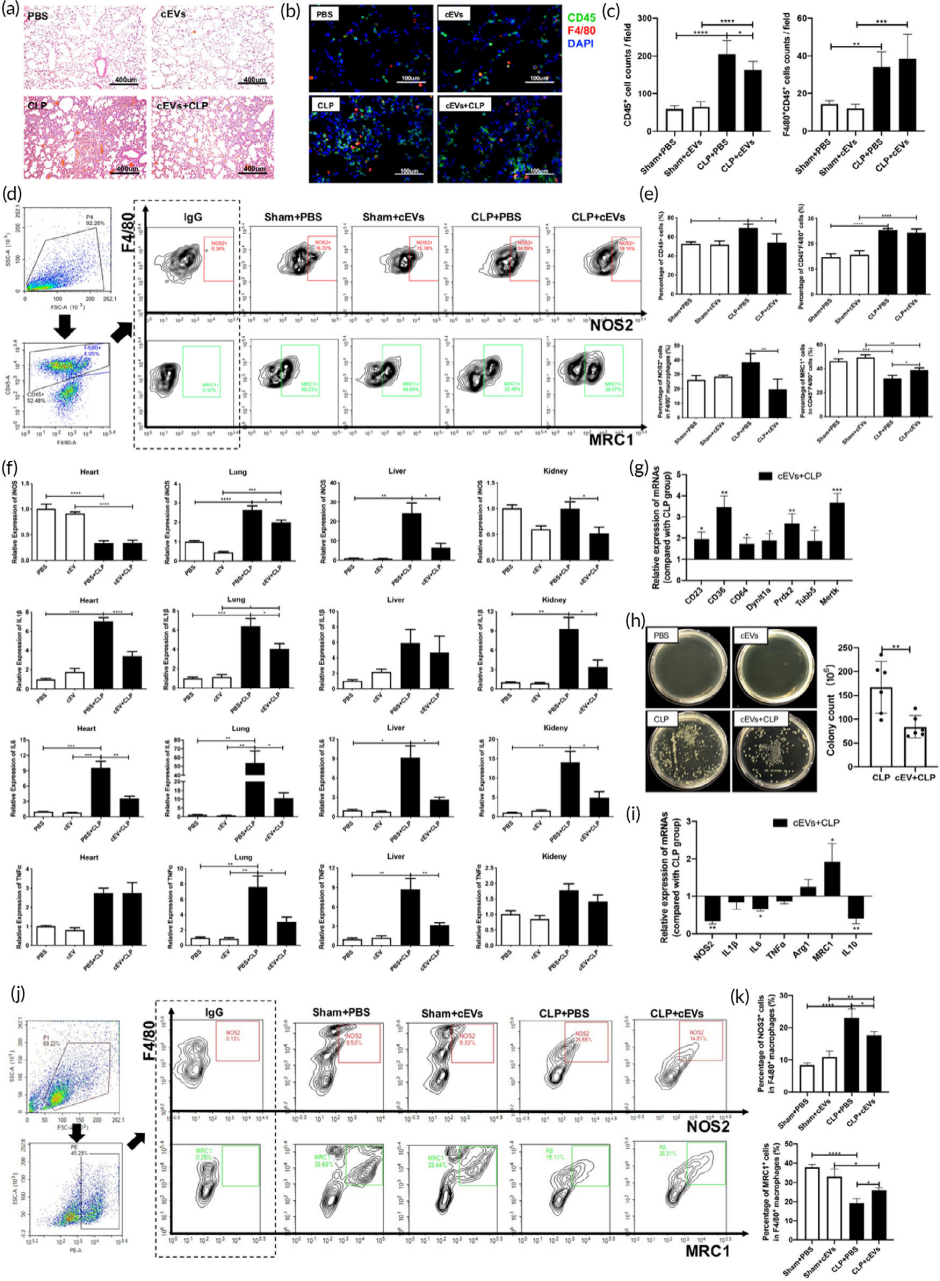
Tissue‐derived EVs attenuate septic injury. Mice were intraperitoneally injected with PBS or cEVs (2 × 10^9^/g mouse) 1 day before CLP. Tissues were harvested 24 h after CLP. (a) Representative H&E staining of the lung. (b) Immunofluorescence images showing the CD45^+^ inflammatory cells and CD45^+^F4/80^+^ macrophages in the lung tissues. (c) Statistical results of CD45^+^ and CD45^+^F4/80^+^ cells (cell counts per field) in the lung immunofluorescence images. (d) Polarization of macrophages in the lung determined by flow cytometry. (e) Statistical results showing the percentage of CD45^+^ and CD45^+^F4/80^+^ cells, as well as the percentage of NOS2^+^ and MRC1^+^ macrophages in the lung. (f) The expressions of NOS2, IL1β, IL6, and TNFα in different organs including the heart, lung, liver and kidney. (g) The expressions of phagocytic‐related genes in peritoneal cells of CLP mice. (h) The colony counts of bacteria in the abdominal cavity. (i) The expressions of M1 and M2 polarized genes in the peritoneal cells of CLP mice. (j) Flow cytometry analysis showed the proportions of NOS2^+^ and MRC1^+^ macrophages in peritoneal cells of CLP mice. (k) Statistical results in (j). These data were representative results (*n* = 6) of three repetitions. *p < 0.05; **p < 0.01; ***p < 0.001; ****p < 0.0001.

Given the significant immunomodulation effect of the tissue derived EVs on macrophages in vitro, we hypothesized that these EVs may help control the severe infectious diseases in vivo. We used a murine CLP induced sepsis model which was commonly applied to mimic the fatal infectious disease characterized by uncontrolled inflammatory responses. As expected, intraperitoneal administration of cEVs (2 × 10^9^/g mouse) 1 day before CLP significantly alleviated the lung injury with reduced infiltration of CD45^+^ immune cells in the lungs (Figure 7a–c). Although the number of macrophages was almost the same between CLP and cEVs+CLP group, there were fewer proinflammatory M1 (NOS2^+^) macrophages and more M2 (MRC1^+^) macrophages in the lung tissue of cEVs+CLP mice (Figure 7d,e). cEV treatment reduced the expression of proinflammatory genes in the vital organs of CLP mice, including heart, lung, liver and kidney (Figure 7f). The expression of MRC1 in the liver and IL10 in the heart and liver were upregulated in the cEVs+CLP group compared with the CLP group (Figure S9). Consistently, similar results were also demonstrated in the pEV treatment group (Figures S10 and S11). In addition, the expressions of phagocytic‐related genes in peritoneal cells were significantly increased (Figure 7g) and the colony counts of bacteria markedly decreased in the abdominal cavity (Figure 7h) in CLP mice treated with cEVs. EVs treatment also decreased the expressions of M1 polarized genes (NOS2 and IL6) and increased the levels of M2 polarized genes (MRC1) in the peritoneal cells of CLP mice (Figure 7i). Flow cytometry results further confirmed that administration of cEVs reduced the proportion of NOS2^+^ macrophages and elevated the percentage of MRC1^+^ macrophages in the peritoneal cells of septic mice (Figure 7j, k). These findings indicated that administration of tissue derived EVs promoted the phagocytic function of macrophages, suppressed both local and systemic inflammation, and mitigated the septic injury in murine CLP models.

### 3.8 Tissue‐derived EVs alleviate sepsis through EV‐educated macrophages in CLP models

**FIGURE 8:**
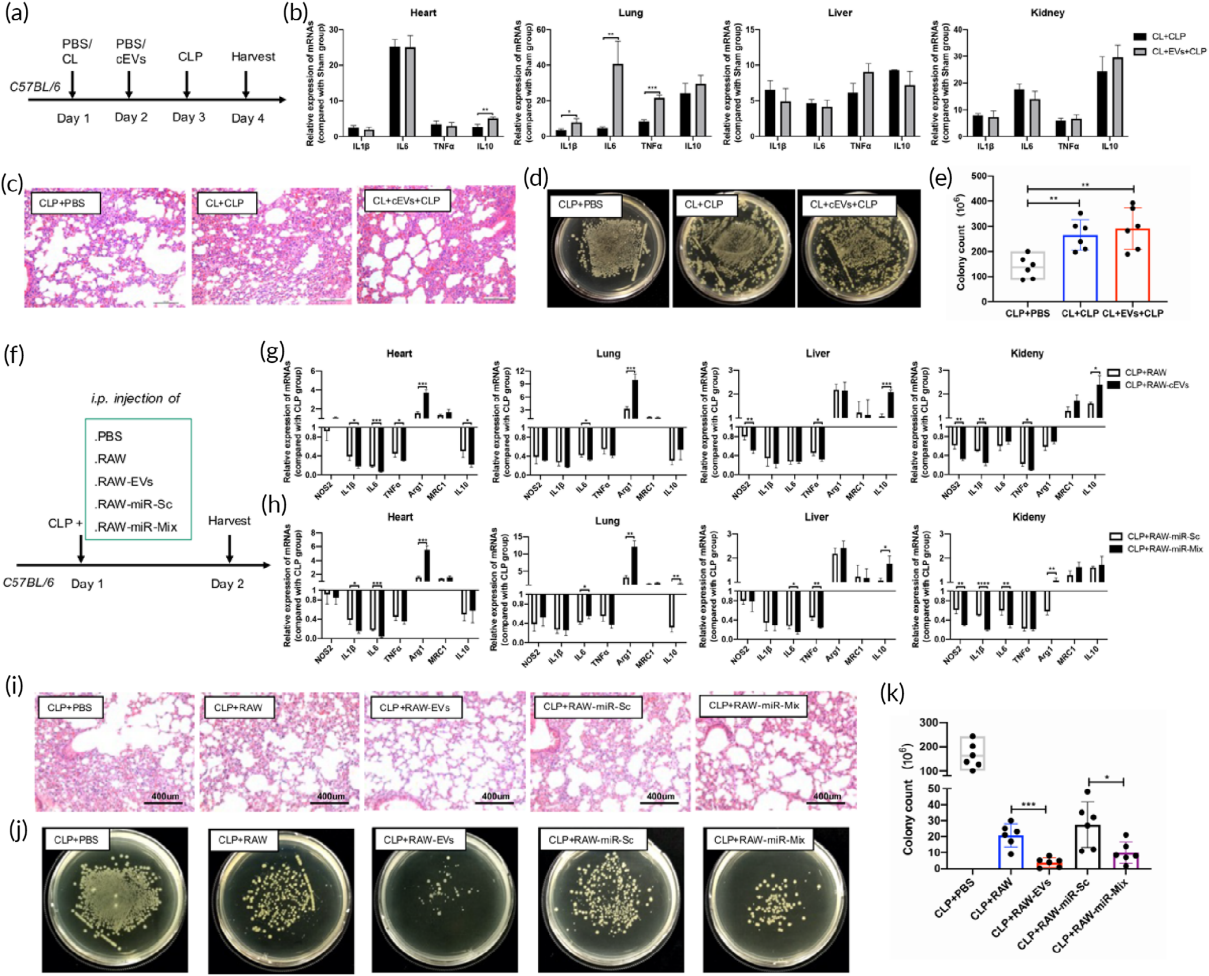
Macrophage is the primary executor in the EV‐mediated sepsis protection. (a) Flowchart illustrated the experimental procedures to investigate the effect of macrophage clearance using clodronate liposomes (CL) on EV‐induced protection in CLP mice, a total of 2 × 10^9^ EVs per gram mouse was used. (b) The expressions of inflammatory cytokines in different organs of the CLP mice in (a). (c) Representative H&E staining of the lung from CLP mice in (a). (d) The colony counts of bacteria in the abdominal cavity. (e) Statistical results in (d). (f) Flowchart illustrated the experimental procedures to investigate the role of EV‐educated macrophages in sepsis. (g) The expressions of M1 and M2 polarized genes in different organs of the CLP mice injected with 2 × 10^6^ RAW264.7 cells (RAW) or cEV‐educated RAW264.7 cells (RAW‐EV). (h) The expressions of M1 and M2 polarized genes in different organs of the CLP mice injected with RAW264.7 (RAW‐miR‐Sc) or RAW264.7 transfected with miRNAs (miR‐148a‐3p, miR‐1a‐3p and miR‐143‐3p) (RAW‐miR‐Mix). (i) Representative H&E staining of the lung tissues. (j) The colony counts of bacteria in the abdominal cavity. (k) Statistical results in (j). These data were representative results (*n* = 6) of three repetitions. *p < 0.05; **p < 0.01; ***p < 0.001; ****p < 0.0001.

To further identify whether EVs exerted their roles in vivo through modulating the functions of macrophages, mice were treated with macrophage scavenger, clodronate liposomes (CL) before EVs‐injection and CLP surgery (Figure 8a). The protective effects of EVs including the inflammation remission (Figure 8b, c) and bacteria removal (Figure 8d, e) in septic mice disappeared after CL treatment. Instead, administration of EVs after macrophage clearance increased inflammatory damage in the lung tissue of septic mice, accompanied by increased (CD45^+^) immune cells, especially (CD45^+^Ly6G^+^) neutrophils in the peritoneal cells of EV‐treated CLP mice (Figure S12).

Furthermore, RAW264.7 macrophages receiving different EV‐treatments were injected into CLP mice (Figure 8f). Macrophage injection significantly reduced the tissue inflammation (Figure 8g), lung injury (Figure 8i), and celiac bacteria burden (Figure 8j,k) in septic mice. More importantly, macrophages treated with EVs protected the mice from septic injury more prominently than those treated with PBS. Consistently, macrophages transfected with EV‐enriched miRNAs (miR‐148a‐3p, miR‐1a‐3p and miR‐143‐3p) showed superior efficacy in CLP mice compared with those transfected with the scramble miRNAs (Figure 8h–k). Therefore, macrophages are supposed to be the primary executor in the EV‐induced protection in murine sepsis models.

## 4 DISCUSSION

Immune homeostasis is the cornerstone of normal life activities. EVs from both non‐immune and immune cells are involved in multiple physiological processes, as well as immune regulation.^14^ The present study for the first time revealed the features of tissue derived EVs from the heart, lung, liver and kidney, and disclosed that EVs from normal tissues can contribute to the maintenance of immune homeostasis and shape the fate of inflammatory diseases.

It is now generally accepted that EVs can be produced by almost all the living cells and exist in all the tissues/organs. Although the role of EVs in health and normal physiology are highlighted recently,^15^ the study of the roles of EVs from different system under healthy conditions is much limited. In this study, we firstly focused on the in vitro features of EVs derived from normal tissues including the heart, lung, liver and kidney. Despite their distinct parent tissues, these EVs are similar in size and shape.

The type and state of the parent cell determine the quantity and content of the EVs it produces.^16^ EV content varies in different tissues, which may attribute to the size of the intercellular space in the tissue as well as the viability of the cells to produce EVs. EVs exert their regulative functions on various immune cells including T cells,^10^ B cells,^17^ monocyte/macrophages^18^ and dendritic cells.^19^ As sentinel cells of the innate immune system, macrophages account for barrier immunity and homeostasis maintenance in various tissues/organs.^20^ A recent study revealed that, in physiological conditions, cardiac macrophages protected the heart from metabolic disorders and ventricular dysfunction by uptaking the dysfunctional mitochondria ejected by cardiomyocytes.^21^ Alveolar macrophages can conceal bacteria from the immune system and contribute to lung homeostasis.^22^ Kupffer cells, microglia and intestine macrophages are also reported to be key players in the homeostasis of the liver, brain and intestine, respectively.^23^, ^24^, ^25^ On the other hand, tissue environment itself is a major controller that can influence the phenotype and function of macrophages.^26^ Our present study confirmed that tissue EVs induced a special activation state of macrophages with increased expressions of both pro‐ and anti‐inflammation related genes in basic condition. Previous study found that urinary EVs from healthy donors was rich in antimicrobial proteins and peptides and the proteomics results indicated that these EVs act as innate immune effectors in the urinary tract.^27^ Therefore, we speculated that EVs in healthy tissues may act as a vital messenger of intercellular communication and contribute to the tissue local homeostasis. It is now accepted that the state of the source cells and organs determines the function of EVs. Therefore, even in the same tissue, EVs have different chemotactic effects on immune cells under different states. Recent studies highlighted the vital role of efferocytosis in tissue homeostasis, tissue repair and organismal health, and macrophage is the primary executor.^28^ The enhanced phagocytosis and decreased migration activity in EV‐educated macrophages may help alert them to stress signals or injuries and retain macrophages in the tissues to engulf apoptotic /necrotic cells, metabolic waste and pathogens.

Macrophages are involved in pathological conditions through differentiating into different subtypes (typically classified as M1 or M2 polarization).^29^, ^30^ The striking inflammatory response of macrophages initiates the onset of the disease.^31^ While anti‐inflammatory macrophages contribute to immune tolerance, tissue healing and fibrosis.^32^ Therefore, the promotion of anti‐inflammatory polarization in macrophages is regarded as a potential therapeutic approach for refractory inflammatory diseases. As a life‐threatening inflammatory disease, sepsis can trigger excessive inflammation and multiple organ complications. The mortality associated with sepsis remains high (25%–30%) and even increases to 40%–50% in the presence of septic shock.^33^ It is well established that profound activation of innate immune cells especially macrophages play vital roles in the immunopathogenesis of sepsis. Large number of bacteria and the inflammatory storm induced by endotoxin are the key factors that make sepsis fatal. The present study revealed a restricted response to LPS and enhanced phagocytic function in EV‐educated macrophages in vitro. As expected, we further confirmed the protective role of tissue derived EVs in murine sepsis models. These results suggested that tissue derived EVs can exert their role in vivo via controlling the function of macrophages. Therefore, tissue‐EVs as endogenous controllers of immune response may provide new thoughts for using these protective EVs or EV‐educated macrophages as therapeutic approaches in inflammatory diseases.

Ultracentrifugation, as the most important method for exosome extraction, still has the risk of confounding contamination. To ensure the reliability of our results, we performed further experiments using EVs purified by SEC and found that the purified‐EVs consistently increased the expression of cytokines, chemokines, and phagocytic‐related genes in macrophages.

Protein and RNA components in EVs are highlighted as important functional molecules in many studies. The present study disclosed the distinct roles of protein and RNA components in tissue EVs on macrophage modulation, and these components may act synergistically or antagonistically on the same factor or pathway, and ultimately participate in macrophage regulation as well as disease progression. This study mainly focused on the inflammatory regulation of EVs on macrophages. However, whether and how these distinct components of EVs influence other cells or other functions of macrophages remain further investigation.

Different tissues have some common types of stromal cells such as endothelial cells and fibroblasts which may produce EVs with similar amounts and contents. As major contents of EVs, miRNAs regulate gene expression and function in target cells. We disclosed that miR‐148a‐3p, miR‐1a‐3p, and miR‐143‐3p were enriched in all these tissue EVs, and these miRNA cargos can be transferred to macrophages. These findings further explained why EVs from different tissues confer similar effects on macrophages. A previous study demonstrated that miR‐148a‐3p repressed the NF‐κB signaling and decreased the expressions of inflammatory genes.^34^ Moreover, miR‐1a‐3p was identified to negatively control the JNK/MAPK pathway during skeletal muscle development.^35^ Overexpression of miR‐143‐3p significantly inhibited the migration of tumor cells.^36^ In this study, the miR‐143‐3p enriched EVs reduced the expression of CCR2 and restrained the migration of macrophages. Multiple pathways were involved in the LPS resistance of EV‐educated macrophages including STAT, NF‐κB (p‐P65/P65), and JNK, and miR‐143‐3p was the most important suppressor of these pathway proteins in our results. A previous study revealed that endothelium derived EVs suppressed the LPS response of monocyte.^37^ EVs from tissue can be released by various parent cells in which endothelial cells represent one of the major populations in these organs. Consistently, we also found that miR‐148a‐3p, miR‐1a‐3p, and miR‐143‐3p are expressed in endothelium‐derived EVs (data not shown). Hence, these tissue derived EVs may mainly come from the local endothelial cells and the anti‐inflammatory effect on macrophages attribute mainly to these enriched miRNAs.

Our study still has some potential limitations. First, the EV‐enriched miRNAs including miR‐148a‐3p, miR‐1a‐3p, and miR‐143‐3p were found based on RNA‐seq using cardiac EVs. Further analysis to evaluate the tissue specific miRNA are needed since the miRNAs content in EVs may vary between different tissues. Second, although we confirmed the role of miR‐148a, miR‐1a, and miR‐143 on macrophage function, the mechanism in which these miRNAs exert their role is not fully described. Thirdly, cell‐to‐cell communication is a delicate and complex process. This study revealed some universal roles of EVs from different tissues. Whether EVs with different origins exert their unique functions on different tissue/organ needs further exploration. Most importantly, in the present study, we evaluated the protective role of EVs from vital organs in fatal inflammatory disease, and efforts to determine whether EVs from healthy allogeneic or heterogeneous organs have the same efficacy may achieve the translational therapeutic aim.

**FIGURE 9:**
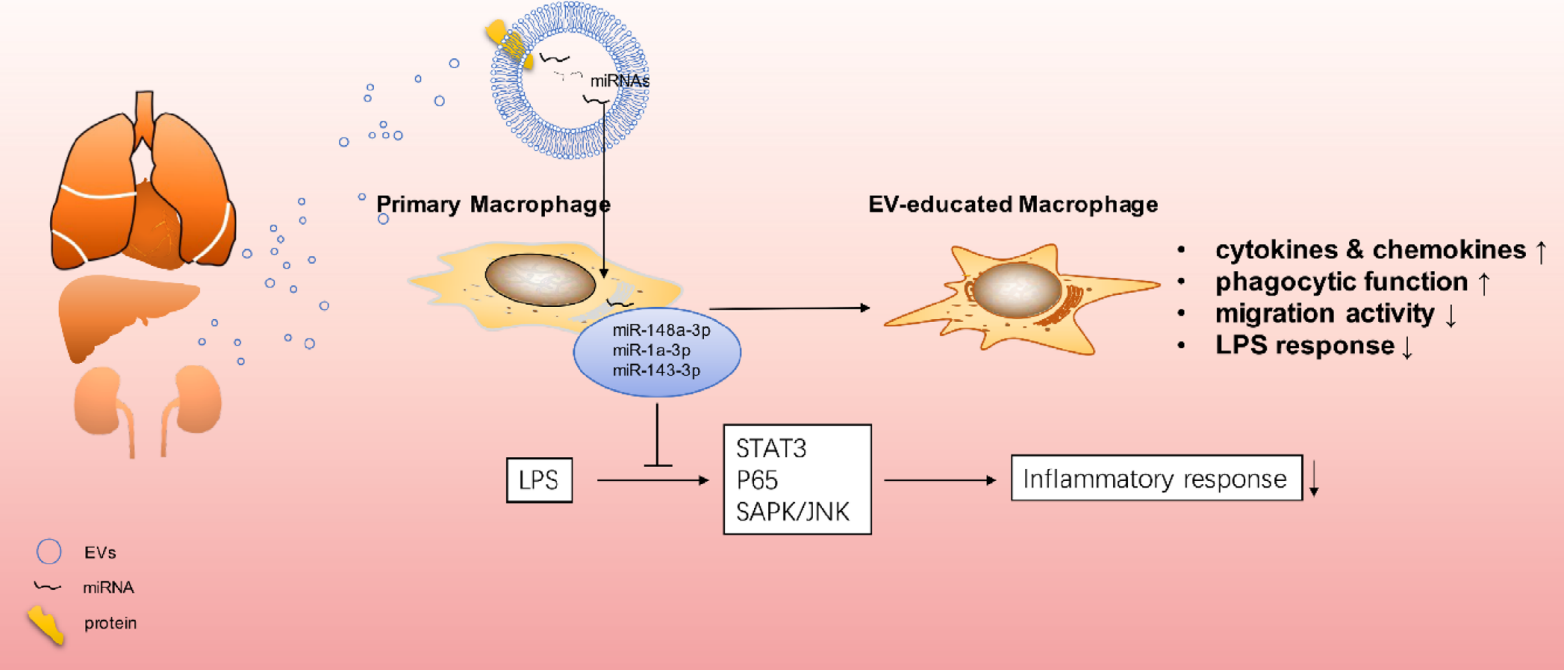
Schematic diagram of the role of EVs from normal tissues on the homeostasis of macrophages. EVs, isolated from murine heart, lung, liver, and kidney have similar effects on macrophages and regulate the inflammation, chemotaxis, and phagocytosis of macrophages. EV‐treated macrophages show LPS resistance with reduced expressions of inflammatory cytokines and enhanced phagocytic activity. EV‐enriched miRNAs, including miR‐148a‐3p, miR‐1a‐3p, and miR‐143‐3p contribute to the inflammation remission in LPS induced macrophages through multiple pathways, including STAT3, P65, and SAPK/JNK.

In summary, the present study disclosed a universal role of EVs from different tissues on macrophage modulation and confirmed the protective role of EVs from healthy tissues in sepsis progression (Figure 9). These findings shed some new lights on the role of tissue EVs in the physio‐pathologic process, supporting the therapeutic potential of the tissue derived EVs or EV‐modulated macrophages in inflammatory diseases.

## AUTHOR CONTRIBUTIONS

**Xinyu Ge:** Data curation (lead); formal analysis (lead); funding acquisition (supporting); investigation (lead); methodology (lead); software (lead); validation (lead); writing – original draft (lead). **Qingshu Meng:** Data curation (equal); formal analysis (equal); investigation (equal); methodology (equal); resources (equal); software (equal). **Xuan Liu:** Data curation (equal); formal analysis (equal); investigation (equal); methodology (equal); validation (equal). **Shanshan Shi:** Data curation (equal); formal analysis (equal); investigation (equal); methodology (equal); validation (equal). **Xuedi Geng:** Formal analysis (equal); methodology (equal). **Enhao Wang:** Investigation (equal); methodology (equal). **Mimi Li:** Data curation (equal); methodology (equal). **Xiaoxue Ma:** Data curation (equal); formal analysis (equal). **Fang Lin:** Methodology (equal); resources (equal); software (equal). **Qianqian Zhang:** Investigation (equal); methodology (equal). **Yinzhen Li:** Data curation (equal); investigation (equal). **Lunxian Tang:** Funding acquisition (equal); resources (equal). **Xiaohui Zhou:** Conceptualization (lead); data curation (equal); formal analysis (equal); funding acquisition (lead); project administration (lead); resources (lead); supervision (lead); writing – review and editing (lead).

## FUNDING INFORMATION

The present study was supported by the National Natural Science Foundation of China (NSFC; grant nos. 81670458, 81970072, and 82100350) and the program of Science and Technology Commission of Shanghai Municipality (STCSM; grant no. 23ZR1452500).

## CONFLICT OF INTEREST

The author declares no conflict of interests.

### PEER REVIEW

The peer review history for this article is available at https://www.webofscience.com/api/gateway/wos/peer-review/10.1002/btm2.10609.

## Supporting information


**DATA S1.** Supporting Information

## Data Availability

Data are available from the corresponding author upon reasonable request.
