# Supplementary material for: Extracellular vesicles from normal tissues orchestrate the homeostasis of macrophages and attenuate inflammatory injury of sepsis
Source: Bioeng Transl Med. 2023 Oct 14;9(1):e10609. doi: 10.1002/btm2.10609 (PMC10771551; doi:10.1002/btm2.10609)
Supplement: Supplementary file 1 — DATA S1. Supporting Information [file BTM2-9-e10609-s001.docx]

Supplementary material

**Extracellular vesicles from normal tissues** **orchestrate the homeostasis of macrophages and** **attenuate inflammatory injury of sepsis**

Xinyu Ge^1-3^, Qingshu Meng^1,2^, Xuan Liu^1-3^, Shanshan Shi^1,2^, Xuedi Geng^1,2^, Enhao Wang^1,2^, Mimi Li^1,2^, Xiaoxue Ma^1,2^, Fang Lin^1,2^, Qianqian Zhang^1,4^, Yinzhen Li^1,5^, Lunxian Tang^4^, Xiaohui Zhou^1,2*^

1. Research Center for Translational Medicine, Shanghai East Hospital, Tongji University School of Medicine, Shanghai 200120, P.R. China;
2. Shanghai Heart Failure Research Center, Shanghai East Hospital, Tongji University School of Medicine, Shanghai 200120, P.R. China;
3. Department of thoracic Surgery, Shanghai East Hospital, Tongji University School of Medicine, Shanghai 200120, P.R. China;
4. Department of Internal Emergency Medicine and Critical Care, Shanghai East Hospital, Tongji University School of Medicine, Shanghai 200120, P.R. China;
5. Department of Respiratory Medicine, Shanghai East Hospital, Tongji University, School of Medicine, Shanghai 200120, P.R. China;

Running title: Tissue EVs contribute to the immune homeostasis

Word count: 8163

* Address for Correspondence:

Xiaohui Zhou, MD, PhD

Shanghai East Hospital, Tongji University School of Medicine, No. 150 Jimo Rd., Shanghai 200120, P.R. China. Tel: 0086-2161569884; Fax: 0086-2158798999; Email: [zxh100@tongji.edu.cn](mailto:zxh100@tongji.edu.cn)

**Supplementary methods**

**Flow cytometry detection of macrophages in lung tissues**

After PBS perfusion from the left ventricle, lung tissues were dissected and transferred into gentleMACS C tube. The tissues were incubated at 37℃ in an enzyme cocktail of RPMI containing 2 mg/ml collagenase I and 40 U/ml DNase (sigma) for 20 minutes, and then dissociated using GentleMACS Dissociator (Miltenyi Biotec). After shaking at 37℃ for 20 minutes, a second-time dissociation using GentleMACS was conducted, then mashed through a 70-μm nylon cell strainer (BD Falcon). Cells were collected and incubated with fluorescence conjugated CD45, F4/80 and MRC1 antibody (BioLegend) at 4°C 30min. For intracellular staining of NOS2, cells were fixed and permeabilized according to manufacturers’ instructions (BD Biosciences) before antibody incubation. Data were acquired using a GalliosTM flow cytometer (Beckman Coulter) and analyzed with FlowJo-V10 software (Tree Star, Ashland, OR, USA).

**Supplementary** **Table 1.** Quantitative polymerase chain reaction primers

| **Gene** | **Forward primer (5'-3')** | **Reverse primer (5'-3')** |
| --- | --- | --- |
| NOS2 | CACCAAGCTGAACTTGAGCGA | CCATAGGAAAAGACTGCACCGA |
| IL1β | GCAACTGTTCCTGAACTCAACT | ATCTTTTGGGGTCCGTCAACT |
| IL6 | TAGTCCTTCCTACCCCAATTTCC | TTGGTCCTTAGCCACTCCTTC |
| TNFα | AAGCCTGTAGCCCACGTCGTA | GGCACCACTAGTTGGTTGTCTTTG |
| Arg1 | CTGAGAAACGGAACCGCGA | TGCTCTTTGATCTGGCGGA |
| MRC1 | TTCAGCTATTGGACGCGAGG | GAATCTGACACCCAGCGGAA |
| Fizz1 | CCAATCCAGCTAACTATCCCTCC | CCAGTCAACGAGTAAGCACAG |
| Ym1 | CCAAGTGCAGCATGTGTCAG | CCTCTACGTTCCCCAAGTCG |
| IL10 | GCTCTTACTGACTGGCATGAG | CGCAGCTCTAGGAGCATGTG |
| TGFβ | CTCCCGTGGCTTCTAGTGC | GCCTTAGTTTGGACAGGATCTG |
| VEGFα | GGCAGCTTGAGTTAAACGAAC | TGGTGACATGGTTAATCGGTC |
| CCL2 | TTAAAAACCTGGATCGGAACCAA | GCATTAGCTTCAGATTTACGGGT |
| CCL4 | TTCCTGCTGTTTCTCTTACACCT | CTGTCTGCCTCTTTTGGTCAG |
| CXCL1 | CTGGGATTCACCTCAAGAACATC | CAGGGTCAAGGCAAGCCTC |
| CXCL2 | CCAACCACCAGGCTACAGG | GCGTCACACTCAAGCTCTG |
| CCR1 | CTCATGCAGCATAGGAGGCTT | ACATGGCATCACCAAAAATCCA |
| CCR2 | ATCCACGGCATACTATCAACATC | CAAGGCTCACCATCATCGTAG |
| CXCR4 | GACTGGCATAGTCGGCAATG | AGAAGGGGAGTGTGATGACAAA |
| CX3CR1 | GAGTATGACGATTCTGCTGAGG | CAGACCGAACGTGAAGACGAG |
| CD32 | TCACCATCACTGTCCAAGGG | GGAGGATTGTATGGGCTGCT |
| CD36 | TCTCCTAGTAGGCGTGGGTC | CACGGGGTCTCAACCATTCA |
| CD64 | GCAAGTTAGAAGCGATGGCG | ATGCCATGGTCCCACAGTTT |
| Dynlt1a | CCAGCACAGCAAAGTCAACC | GCAAGGTGGTCAGATGGACA |
| Prdx2 | GCAAATCGGAAAGTCGGCTC | CTCCGTGGGGCAAACAAAAG |
| Tubb5 | TGCCCTTTGTCCTCCAGTTT | TAGGAAGCACAGGGTAACAACC |
| Mertk | CGAGCCATCGAGCTTACCTT | ACTCCTCGATCTCCCGTTGA |
| Pri-mir1a | ACCTACCTGCTTGGGACACA | GCGAGAGAGTTCCTAGCCTG |
| Pri-mir143 | TGCTGCATCTCTGGTCAGTT | GCAGGACATCTTCTCCCTTCC |
| Pri-mir148 | AGGAAGACAGCCAGTTTGGTC | CCACAGCCTCTAGAGACAAAGTT |
| β-actin | ACCTTCTACAATGAGCTGCG | CTGGATGGCTACGTACATGG |
| mmu-miR-1a-3p | CGCGCCGCGTGGAATGTAAAGAAGTATGTAT | Universal reverse primer |
| mmu-miR-143-3p | CCGCGTGAGATGAAGCACTGTAGCTC | Universal reverse primer |
| mmu-miR148a-3p | CGGCGTCAGTGCACTACAGAACTTTGT | Universal reverse primer |
| Mouse U6 | TGGCCCCTGCGCAAGGATG | Universal reverse primer |

**Supplemental** Figures

**
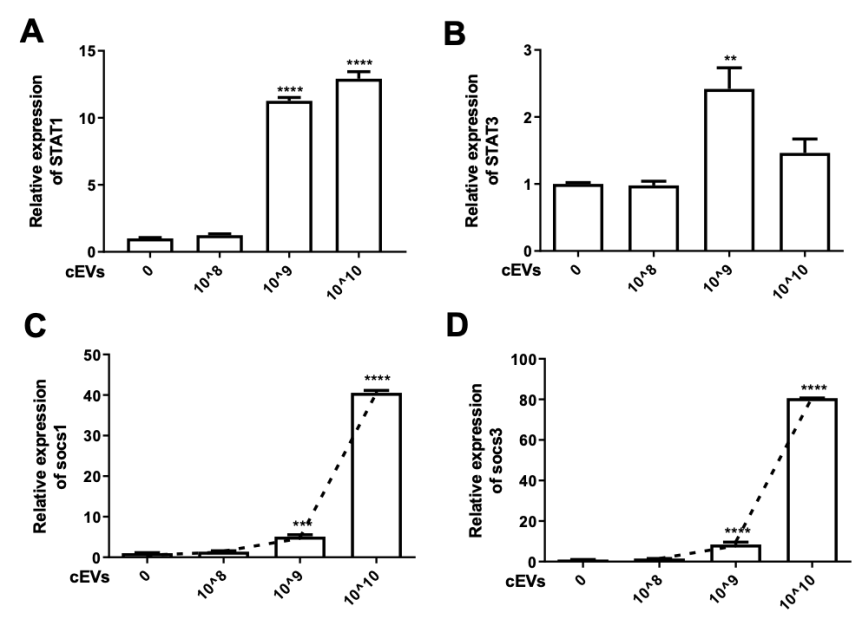
**

**Supplemental Figure 1.** The expressions of inflammation pathway related genes in EVs-treated macrophages. The expressions of **(A)** STAT1, **(B)** STAT3, **(C)** SOCS1 and **(D)** SOCS3 in macrophages treated with gradient concentrations of cardiac EVs. ^*^, P <0.05; ^**^, P <0.01; ^***^, P <0.001; ^****^, P<0.0001.

**
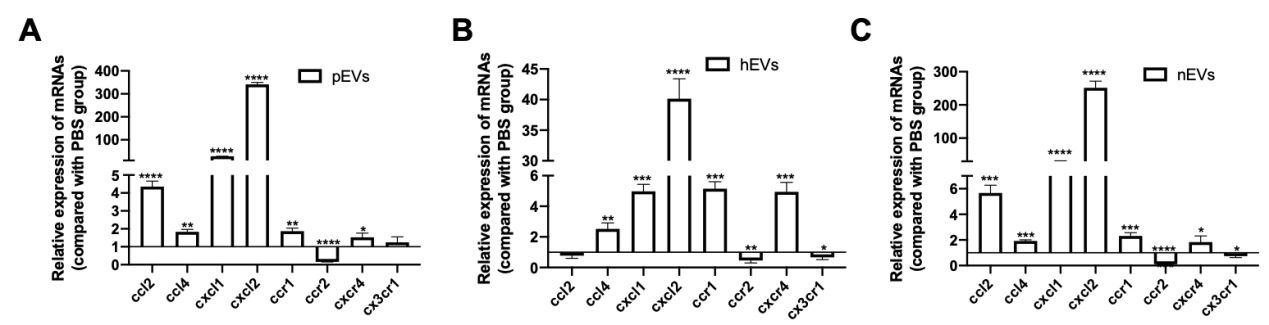
**

**Supplemental Figure 2.** The expressions of chemokines and chemokine receptors in macrophages treated with tissue-derived EVs. The expression of chemokines and chemokine receptors in macrophages treated with **(A)** pulmonary EVs, pEVs, **(B)** hepatic EVs, hEVs, and **(C)** nephritic EVs, nEVs. ^*^, P <0.05; ^**^, P <0.01; ^***^, P <0.001; ^****^, P<0.0001.

**
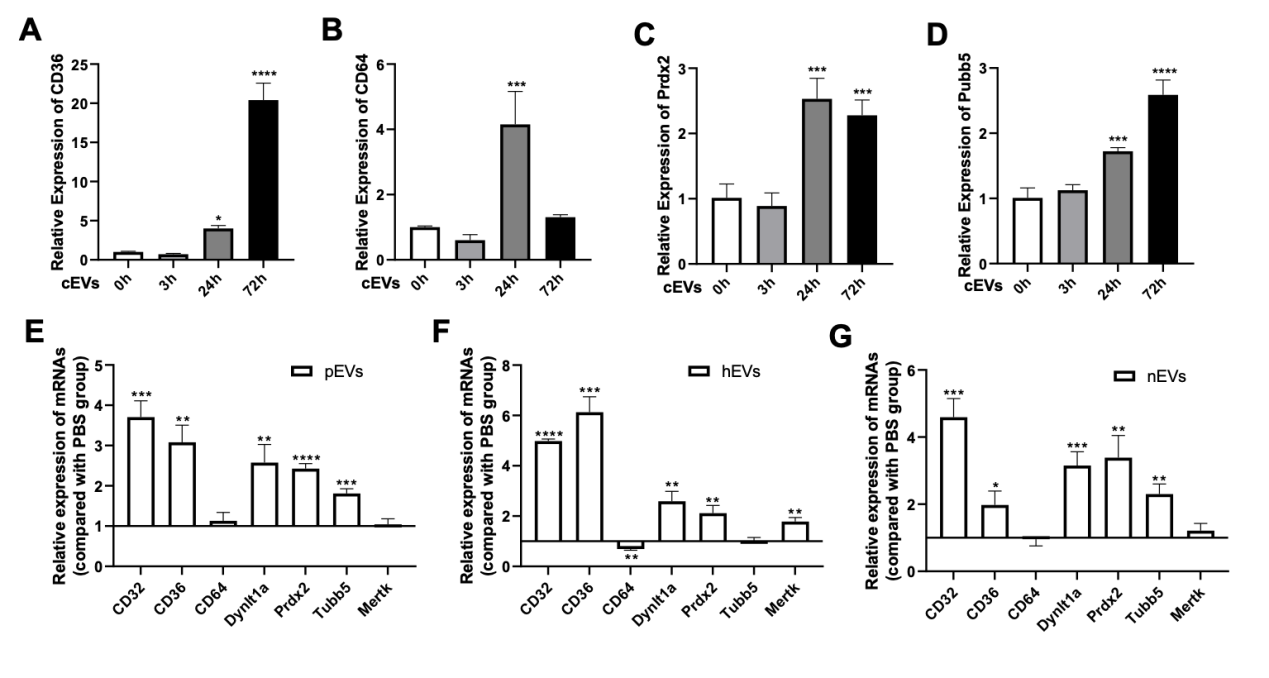
**

**Supplemental Figure 3.** The effect of tissue-derived EVs on macrophage phagocytosis. The expressions of phagocytic-related genes including **(A)** CD36, **(B)** CD64, **(C)** Prdx2 and **(D)** Pubb5 in macrophages treated with cEVs for the indicated time. The expressions of phagocytic-related genes in macrophages treated with **(E)** pEVs, **(F)** hEVs, and **(G)** nEVs for 24h. ^*^, P <0.05; ^**^, P <0.01; ^***^, P <0.001; ^****^, P<0.0001.

**
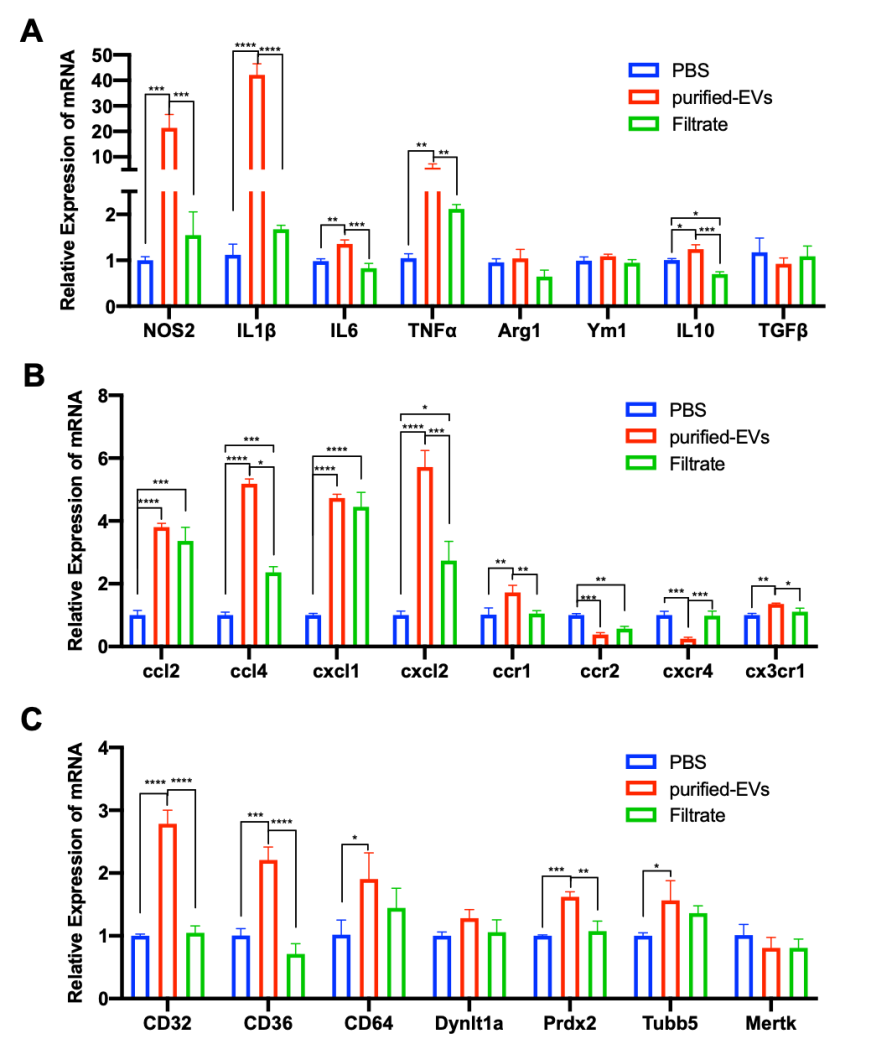
**

**Supplemental Figure 4.** The effect of purified EVs on macrophage. EVs in heart tissues were isolated by ultracentrifugation and purified using size exclusion chromatography (SEC). The purified EVs and filtrate were collected and separately cocultured with macrophages for 24h. The expression of **(A)** M1/M2 polarized genes, **(B)** Chemokines and chemokine receptors, and **(C)** Phagocytosis related genes in macrophages treated with PBS, purified-EVs or EV filtrate. ^*^, P <0.05; ^**^, P <0.01; ^***^, P <0.001; ^****^, P<0.0001.

**
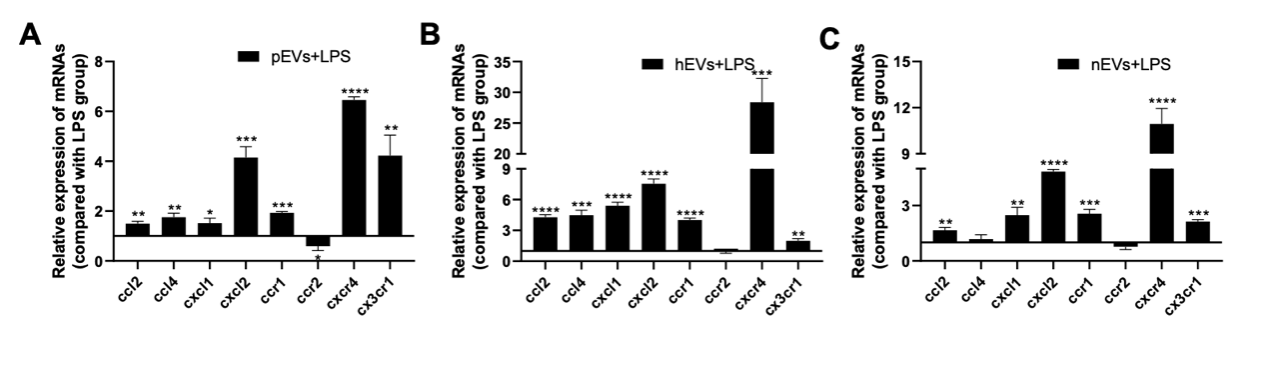
**

**Supplemental Figure 5.** Tissue EVs modulate the chemotactic function of LPS-induced macrophages. Macrophages were pretreated with **(A)** pEVs, **(B)** hEVs, and **(C)** nEVs overnight and then stimulated with LPS for 3h. The expressions of chemokines (ccl2, ccl4, cxcl1 and cxcl2) and chemokine receptors (ccr1, ccr2, cxcr4 and cx3xr1) in macrophages were detected by qPCR. ^*^, P <0.05; ^**^, P <0.01; ^***^, P <0.001; ^****^, P<0.0001.

**
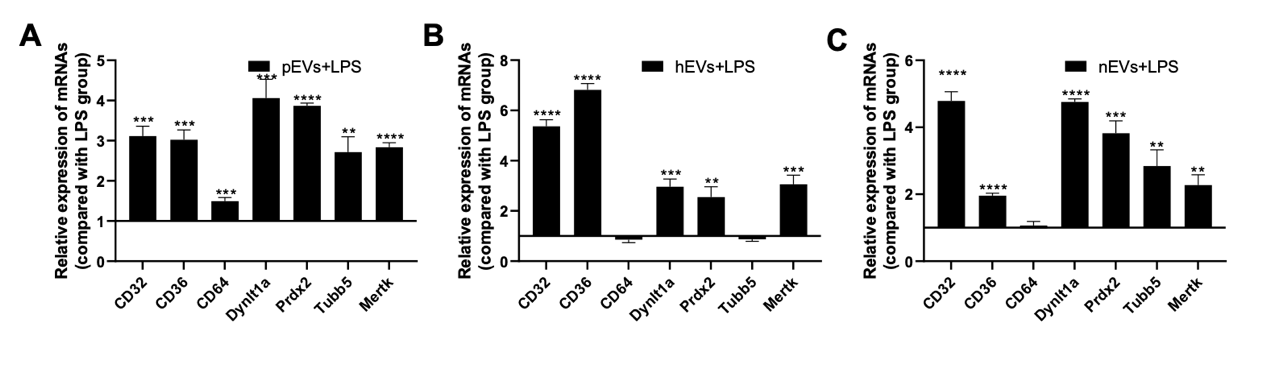
**

**Supplemental Figure 6.** Tissue EVs modulate the phagocytic function of LPS-induced macrophages. Macrophages were pretreated with **(A)** pEVs, **(B)** hEVs, and **(C)** nEVs overnight and then stimulated with LPS for 3h. The expression of phagocytosis related genes in macrophages were detected by qPCR. ^*^, P <0.05; ^**^, P <0.01; ^***^, P <0.001; ^****^, P<0.0001.

**
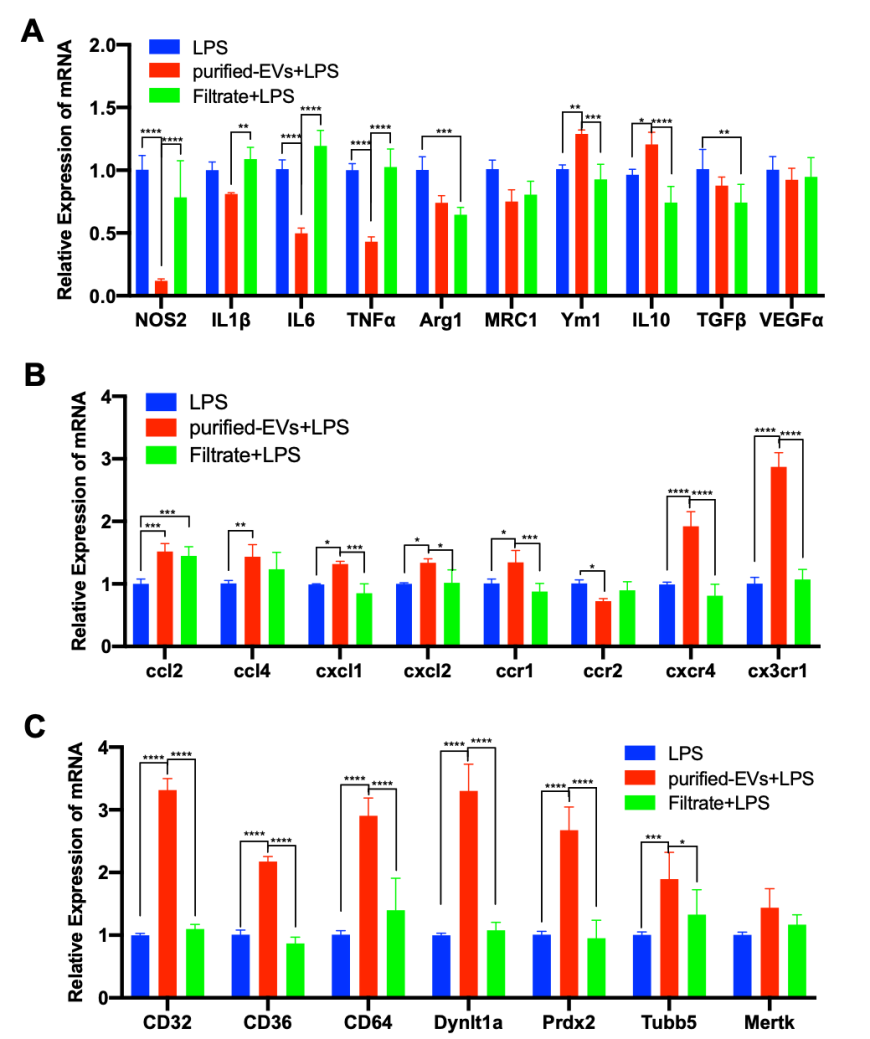
**

**Supplemental Figure 7.** The effect of purified EVs on LPS induced responses in macrophage. Macrophages were treated with purified EVs or filtrate overnight and then stimulated with LPS for 3h. The expressions of **(A)** M1/M2 polarized genes, **(B)** Chemokines and chemokine receptors, **(C)** Phagocytosis related genes in macrophages were detected by qPCR. ^*^, P <0.05; ^**^, P <0.01; ^***^, P <0.001; ^****^, P<0.0001.

**
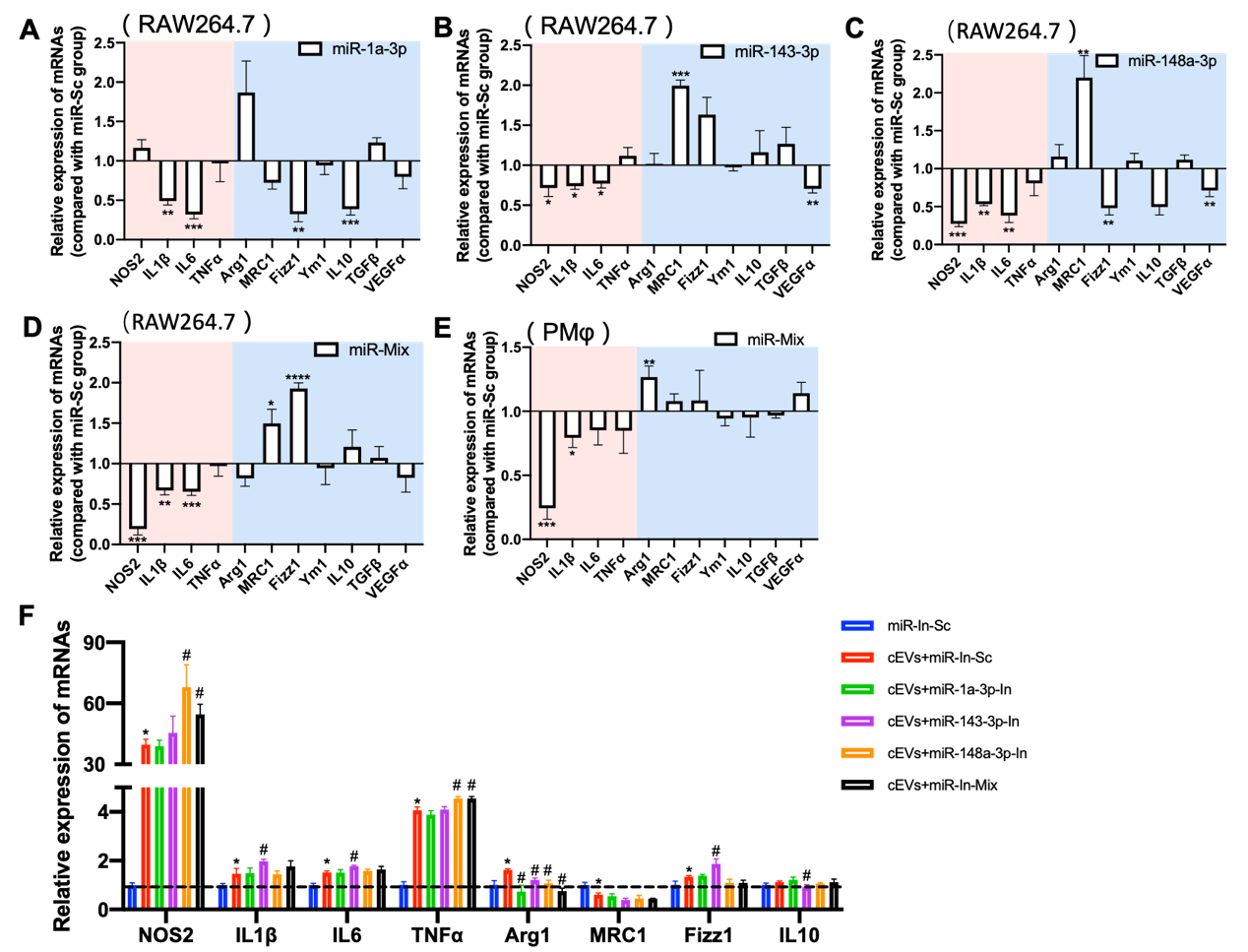
**

**Supplemental Figure 8.** The effect of EV-enriched miRNAs on macrophage polarization. The expressions of M1/M2 polarized genes in macrophage RAW264.7 transfected with **(A)** miR-1a-3p, **(B)** miR-143-3p, **(C)**miR-148a-3p, and **(D)** miRNA-Mix. **(E)** The expressions of M1/M2 polarized genes in peritoneal macrophages (PMφ) transfected with miRNA-Mix. **(F)** Cardiac EVs were transfected overnight with miR-In-Sc (miRNA inhibitor scramble), miR-1a-3p-In (miR-1a-3p inhibitor), miR-143-3p-In (miR-143-3p inhibitor), miR-148a-3p-In (miR-148a-3p inhibitor) or miR-In-Mix (miRNA inhibitor Mix). The transfection system was then separately cultured with RAW264.7 for 24h. qPCR analysis showed the effect of different transfection systems on macrophage polarization. ^*^P < 0.05 vs. miR-In-Sc; ^#^P < 0.05 vs. cEVs+miR-In-Sc.

**
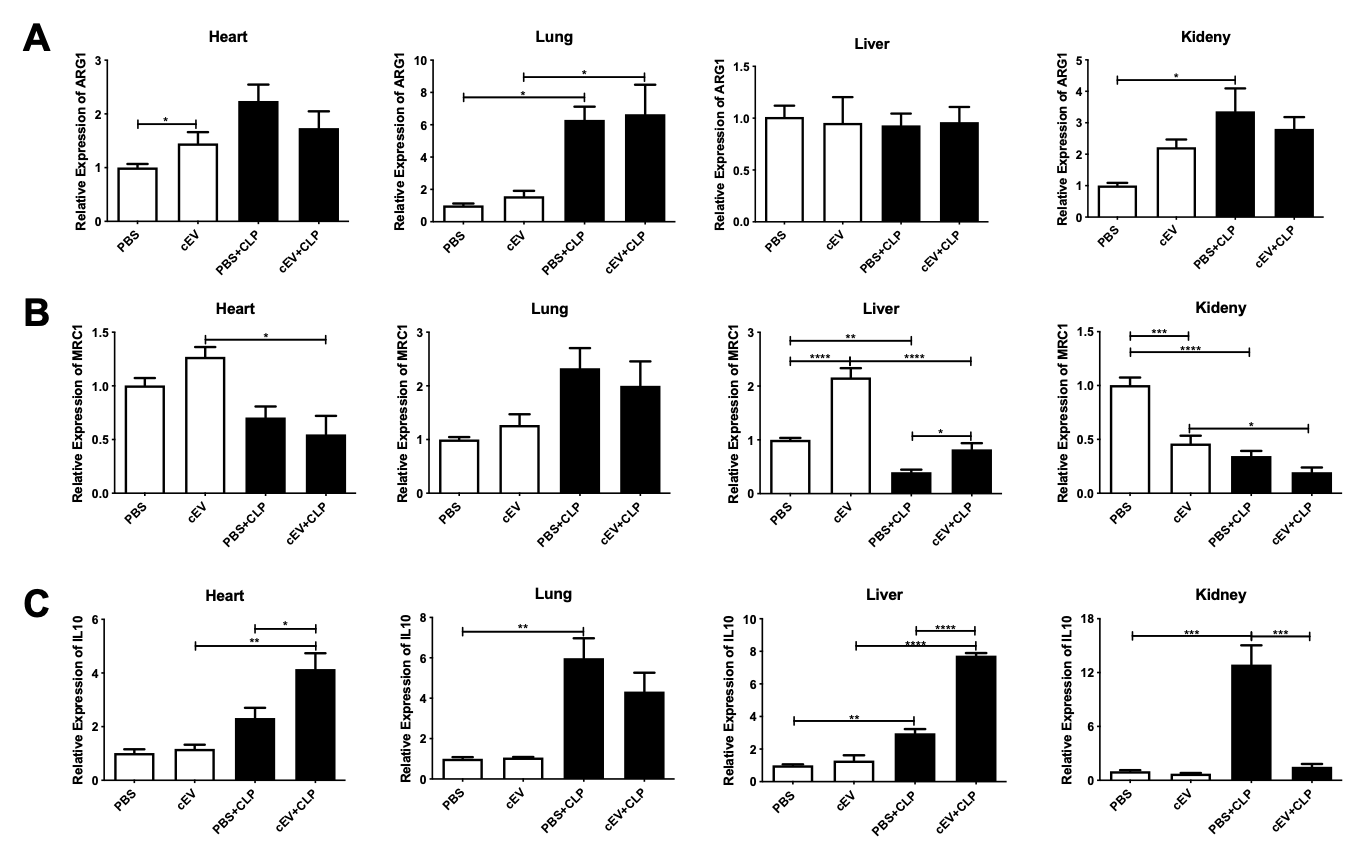
**

**Supplemental Figure 9.** The effect of cEVs on the expressions of anti-inflammatory factors in different organs. Mice were intraperitoneally injected with PBS or cEVs one day before CLP. Tissues were harvested 24h after CLP. The expressions of **(A)** ARG1, **(B)** MRC1, and **(C)** IL10 in different organs including the heart, lung, liver and kidney were detected by qPCR. ^*^, P <0.05; ^**^, P <0.01; ^***^, P <0.001; ^****^, P<0.0001.

**
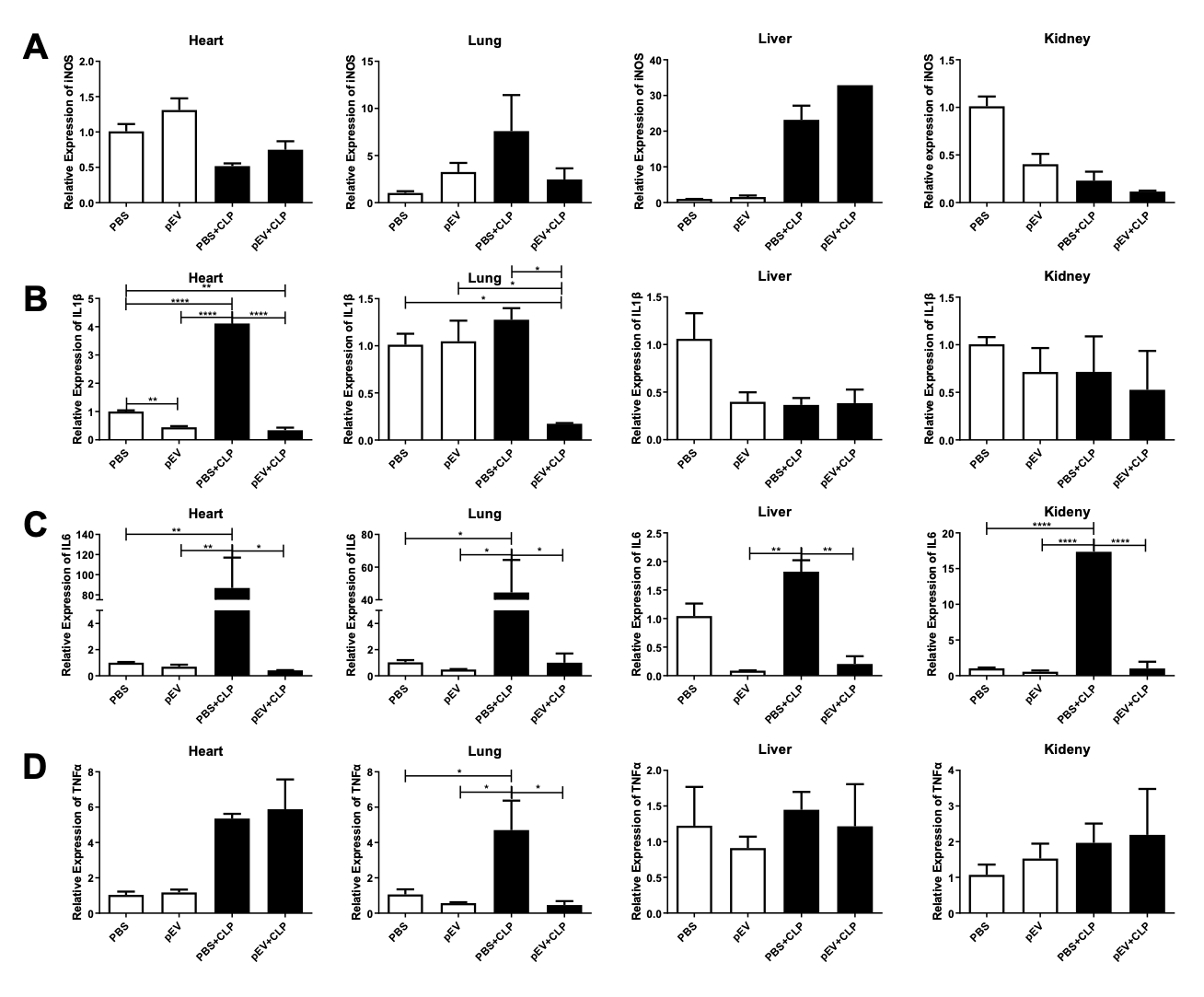
**

**Supplemental Figure 10.** The effect of pEVs on the expressions of proinflammatory factors in different organs. Mice were intraperitoneally injected with PBS or pulmonary EVs (pEVs) one day before CLP. Tissues were harvested 24h after CLP. The expression of **(A)** NOS2, **(B)** IL1β, **(C)** IL6 and **(D)** TNFα in different organs including the heart, lung, liver and kidney were detected by qPCR. ^*^, P <0.05; ^**^, P <0.01; ^***^, P <0.001; ^****^, P<0.0001.

**
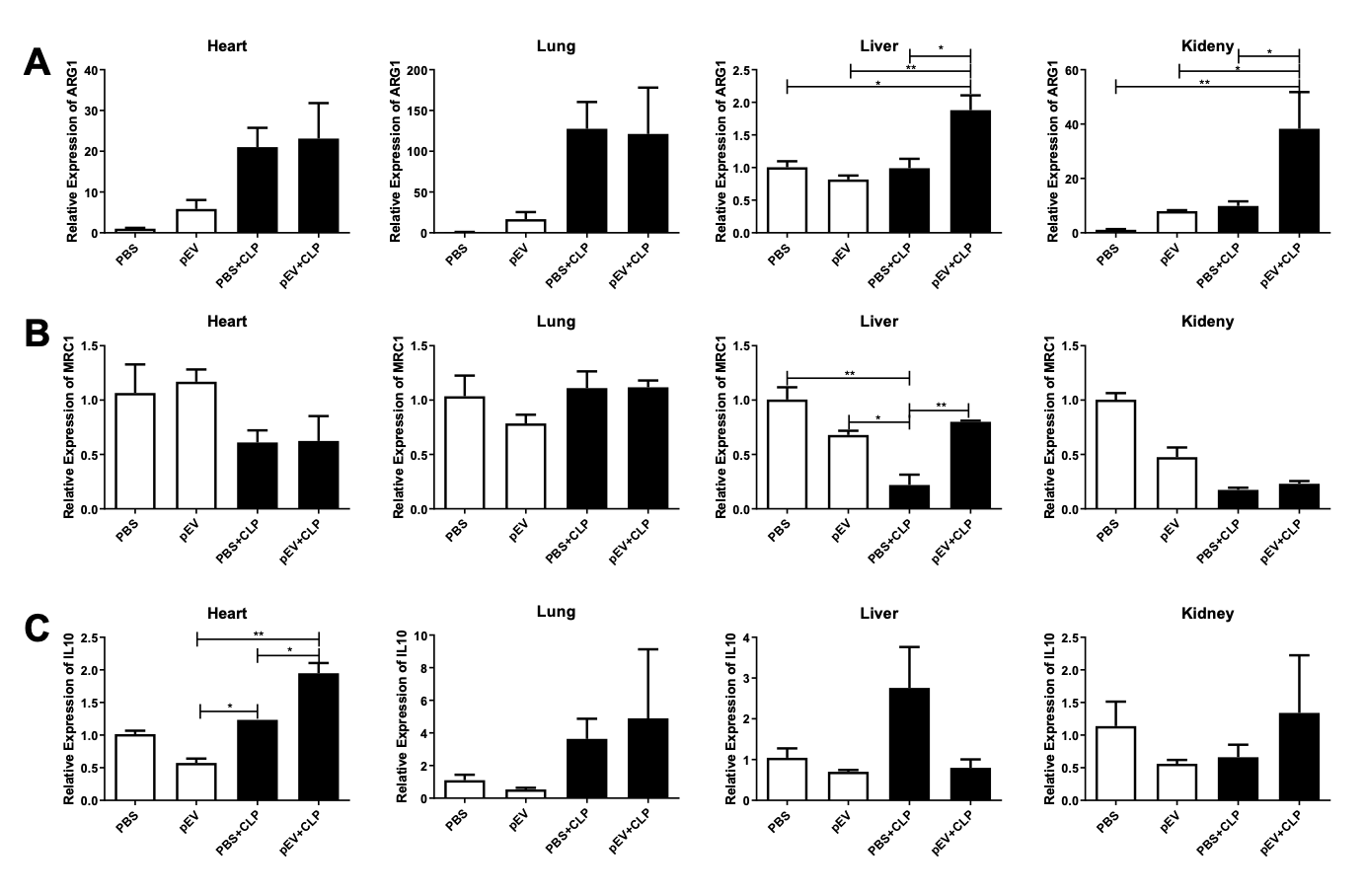
**

**Supplemental Figure 11.** The effect of pEVs on the expressions of anti-inflammatory factors in different organs. Mice were intraperitoneally injected with PBS or pulmonary EVs (pEVs) one day before CLP. Tissues were harvested 24h after CLP. The expression of **(A)** ARG1, **(B)** MRC1, and **(C)** IL10 in different organs including the heart, lung, liver and kidney were detected by qPCR. ^*^, P <0.05; ^**^, P <0.01; ^***^, P <0.001; ^****^, P<0.0001.


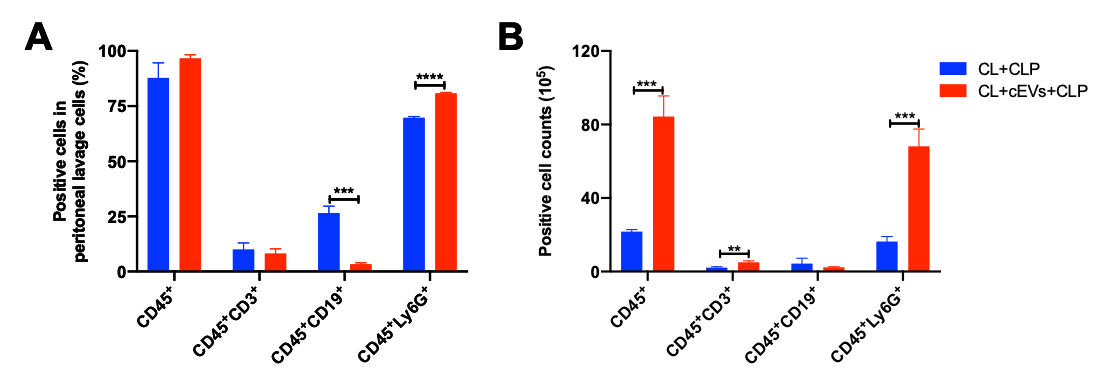


**Supplemental Figure 12.** The effects of cardiac EVs on the composition of immune cells in peritoneal cells. Mice were intraperitoneally injected with clodronate liposomes (CL) for 24h, and cardiac EVs were then injected one day before CLP. Tissues were harvested 24h after CLP. The (CD45^+^) immune cells, including (CD45^+^CD3^+^) T cells, (CD45^+^CD19^+^) B cells and (CD45^+^Ly6G^+^) neutrophils in the peritoneal cells were detected by flow cytometry. ^*^, P <0.05; ^**^, P <0.01; ^***^, P <0.001; ^****^, P<0.0001.
